# Artificial intelligence in risk prediction and diagnosis of vertebral fractures

**DOI:** 10.1038/s41598-024-75628-2

**Published:** 2024-12-19

**Authors:** Srikar R. Namireddy, Saran S. Gill, Amaan Peerbhai, Abith G. Kamath, Daniele S. C. Ramsay, Hariharan Subbiah Ponniah, Ahmed Salih, Dragan Jankovic, Darius Kalasauskas, Jonathan Neuhoff, Andreas Kramer, Salvatore Russo, Santhosh G. Thavarajasingam

**Affiliations:** 1 Imperial Brain & Spine Initiative, Imperial College London, London, UK; 2 Faculty of Medicine, Imperial College London, London, UK; 3 Department of Neurosurgery, University Medical Center Mainz, Langenbeckstraße 1, Mainz, Germany; 4 Center for Spinal Surgery and Neurotraumatology, Berufsgenossenschaftliche Unfallklinik Frankfurt am Main, Frankfurt, Germany; 5 Department of Neurosurgery, Imperial College Healthcare NHS Trust, London, UK

**Keywords:** Artificial intelligence, Machine learning, Osteoporotic vertebral fractures, Non-pathological vertebral fractures, Vertebral compression fractures, ML, AI, OF, VF, Bone, Outcomes research

## Abstract

With the increasing prevalence of vertebral fractures, accurate diagnosis and prognostication are essential. This study assesses the effectiveness of AI in diagnosing and predicting vertebral fractures through a systematic review and meta-analysis. A comprehensive search across major databases selected studies utilizing AI for vertebral fracture diagnosis or prognosis. Out of 14,161 studies initially identified, 79 were included, with 40 undergoing meta-analysis. Diagnostic models were stratified by pathology: non-pathological vertebral fractures, osteoporotic vertebral fractures, and vertebral compression fractures. The primary outcome measure was AUROC. AI showed high accuracy in diagnosing and predicting vertebral fractures: predictive AUROC = 0.82, osteoporotic vertebral fracture diagnosis AUROC = 0.92, non-pathological vertebral fracture diagnosis AUROC = 0.85, and vertebral compression fracture diagnosis AUROC = 0.87, all significant (p < 0.001). Traditional models had the highest median AUROC (0.90) for fracture prediction, while deep learning models excelled in diagnosing all fracture types. High heterogeneity (I² > 99%, p < 0.001) indicated significant variation in model design and performance. AI technologies show considerable promise in improving the diagnosis and prognostication of vertebral fractures, with high accuracy. However, observed heterogeneity and study biases necessitate further research. Future efforts should focus on standardizing AI models and validating them across diverse datasets to ensure clinical utility.

**Supplementary Information:**

The online version contains supplementary material available at 10.1038/s41598-024-75628-2.

## Introduction

Vertebral fractures, as the most frequent type of fragility fractures, are a hallmark of osteoporosis, particularly among the elderly. Studies in Europe show that for individuals aged 50 and older, the incidence rates of new vertebral fractures stand at 10.7 per 1000 person-years for women and 5.7 per 1000 person-years for men^1,2^. Globally, they can account for up to 8.6 million cases per year^3^. Risk factors include inactivity, chronic conditions (such as osteoporosis), smoking and previous falls^4,5^. With the rate of osteoporosis reported to be rising^6^, the subsequent incidence of vertebral fractures is also predicted to increase. Vertebral fractures, unlike fractures of other areas of the skeleton, tend not to be treated at the time of injury, with up to 33% going undetected^7,8^. This results in an increased risk of mortality after such injuries^9^, and can lead to chronic pain and disability in the long term, with significant economic ramifications^10^. As such, the timely detection and treatment of vertebral fractures has become a key challenge for healthcare providers.

While Artificial Intelligence (AI), including its subset Machine Learning (ML), is no longer a novel concept, the rise in its clinical usage has been exponential in recent years^11–13^. Multimodal data, along with the development of the ethical framework surrounding AI, have had an impact in the uptake of AI within the medical field^14^. Diagnostically, AI based systems are currently being used, and have potential, to speed up and improve the precision in diagnostic medicine^15^. Clinically, AI models have been used heavily within dermatology, orthopaedics, and otorhinolaryngology demonstrate the utility of such models in different medical specialties^16–18^. However, the uptake of AI in clinical spinal neurosurgery has been less pronounced.

The current approach to diagnosing and classifying vertebral fractures involves different members of a multidisciplinary team, including specialists from orthopaedics, radiology, neurosurgery, and, in some cases rheumatology and geriatrics. The combined clinical experience can often be limited by intrinsic risks of inaccuracies and lack of efficiency. As such, the use of AI, with a focus on Machine Learning, in these situations is of significant interest^19,20^. However, a robust analysis including both qualitative and quantitative synthesis is required evaluate its use in this context – however such an analysis does not exist currently. Hence, this systematic review aims to assess the literature surrounding the use of AI, particularly Machine Learning, in the detection and prognostication of vertebral fractures.

## Methodology

### Literature search strategy

**Figure. 1:**
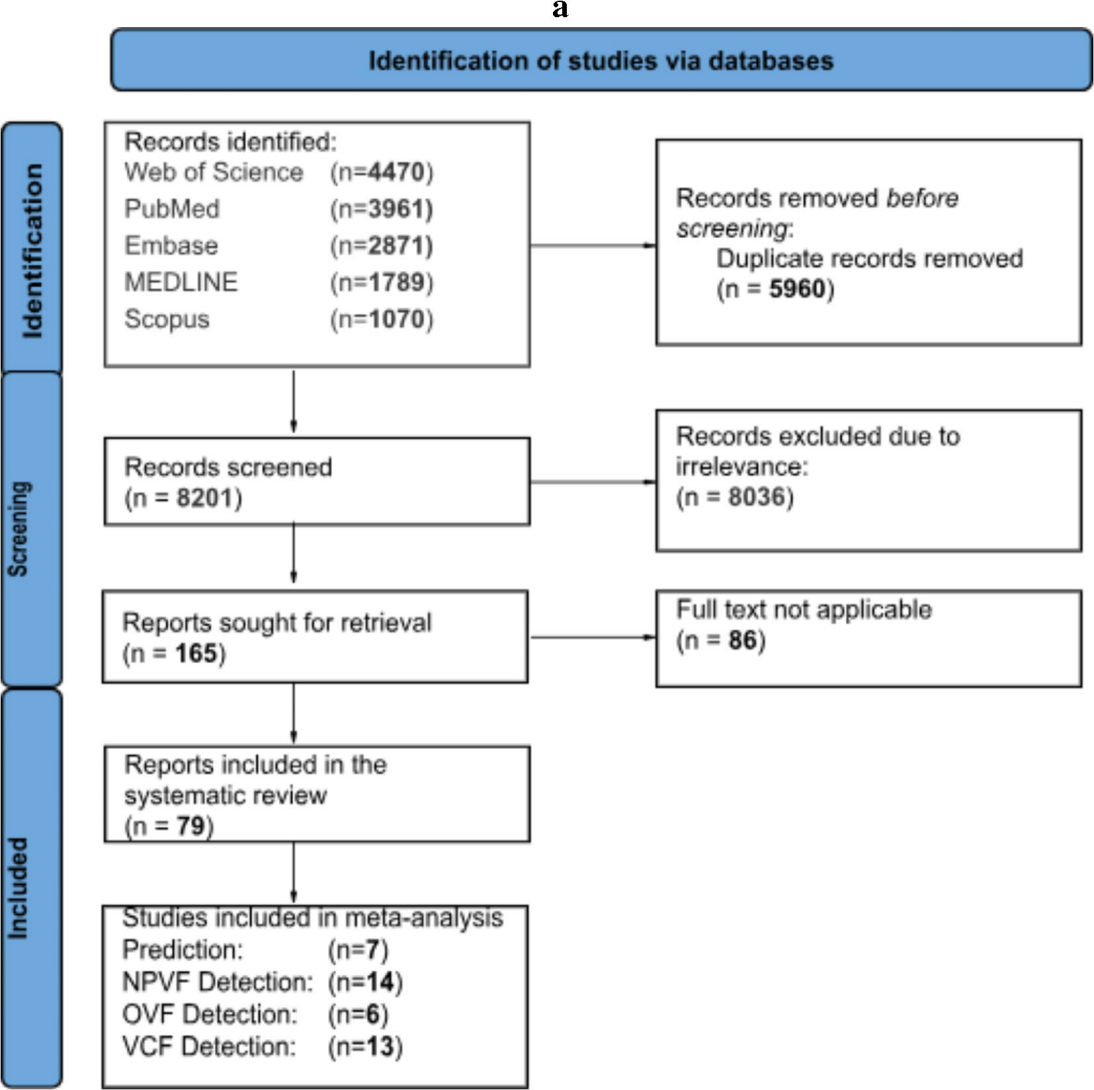

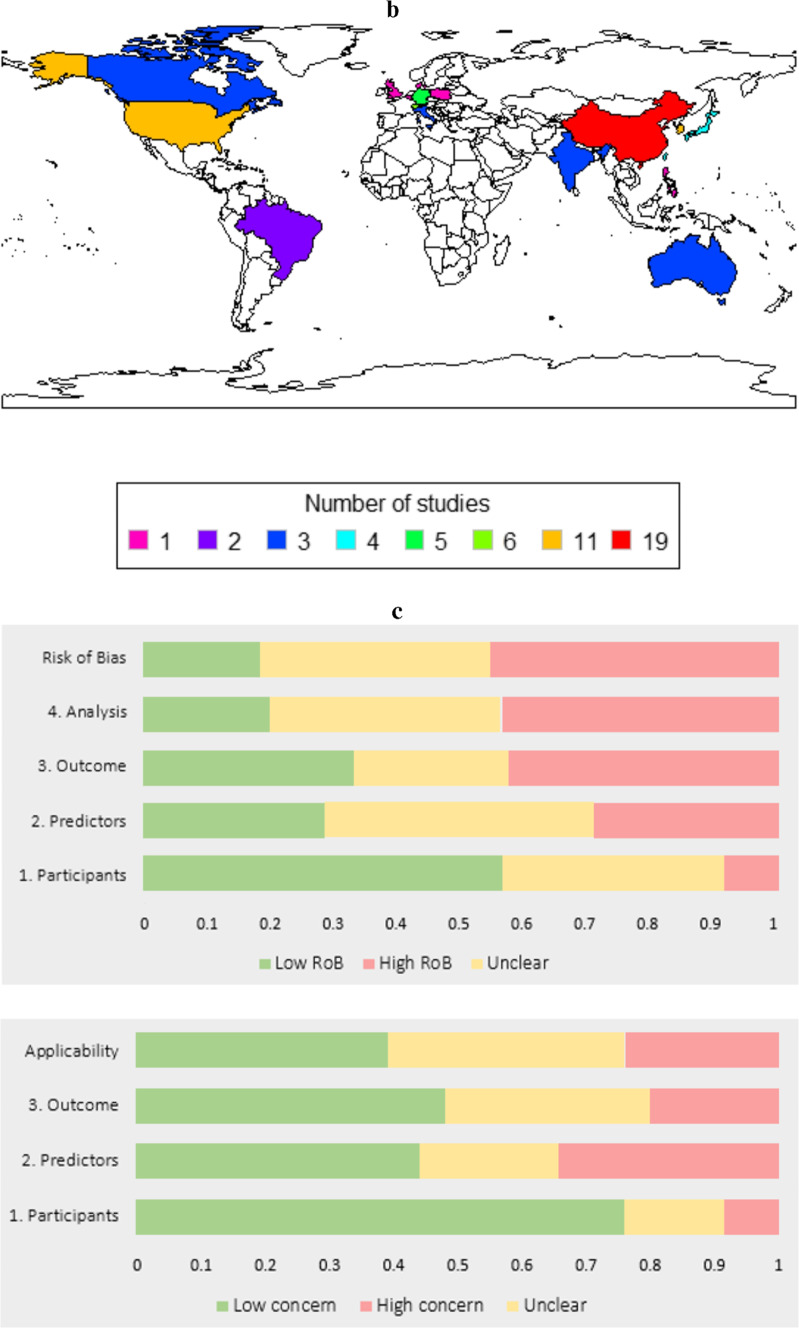
** a**. The preferred Reporting Items for Systematic Reviews and Meta-Analyses (PRISMA) flowchart outlining the study selection process is shown. **b**. A world map indicated the origin of publications included in this study (n = 79) (Superscript references). The countries are coloured according to whether n = 1, 2, 3, 4, 5, 6, 11 or 19 studies from these countries have been included in this systematic review. Following countries are coloured: Germany (n = 5), China (n = 19), South Korea (n = 11), United States of America (n = 11), Brazil (n = 2), Japan (n = 4), Taiwan (n = 4), Italy (n = 3), Canada (n = 3), Switzerland (n = 6), Denmark (n = 1), India (n = 3), Australia (n = 3), Belgium (n = 1), Philippines (n = 1), Poland (n = 1), United Kingdom (n = 1). This map was created using R software (version 4.3.0; https://www.r-project.org/ ) with the rworldmap and ggplot2 packages. **c**. A risk of bias summary plot for all included studies (n = 79) across the domains of the Prediction model Risk Of Bias Assessment Tool (PROBAST).

This systematic review was conducted using the guidelines outlined by the Cochrane Collaboration, and the Preferred Reporting Items for Systematic Reviews and Meta-Analyses (PRISMA). The detailed study protocol can be found in Supplemental Digital Content 1: Supplementary Material S1. The completed PRISMA flowchart is shown in Fig. 1a. The literature search was carried out on February 12th, 2024, using a search of MEDLINE, Embase, Scopus, PubMed, and Web of Science Library. Search strings were created for the following research question: “Is AI an effective and accurate tool for predicting and diagnosis vertebral fractures?”. The search string can be found in Supplemental Digital Content 1: Supplementary Table S1.

### Inclusion and exclusion criteria

The inclusion and exclusion criteria can be found in Supplemental Digital Content 1: Supplementary Table S2. Vertebral fractures were defined as the breakage or collapse of one or more bones in the spine, often leading to pain, reduced mobility, and potential changes in posture^2^. Only studies that used artificial intelligence tools for the diagnostic and prognostication of vertebral fractures were included in the meta-analysis.

### Screening and appraisal

Identified studies were uploaded to COVIDENCE for duplicate removal and title and abstract screening. In the first abstract screening, conducted by four reviewers (SG, AGK, SRN, AP). All original articles in the English language that reported on vertebral fractures were included. Subsequently, only studies reporting on artificial intelligence tools for diagnosis and/or prognostication which also fulfilled our inclusion criteria were included. All included papers were assessed by two independent reviewers. Any disagreements were resolved by consensus after discussion with SRN and HSP.

### Critical appraisal

Two evaluators independently used the Prediction model Risk Of Bias Assessment Tool (PROBAST) to gauge potential biases in the studies analysed^21^. PROBAST examines four key aspects: participants, predictors, outcomes, and analysis. Within these areas, biases related to participant selection, prediction methods, outcome determination, and data analysis were scrutinized using specific guiding questions. Discrepancies in study quality were resolved by a third reviewer. In our review, adherence to the Transparent Reporting of a multivariable prediction model for Individual Prognosis Or Diagnosis (TRIPOD) guidelines was rigorously evaluated by two independent researchers for each included study. TRIPOD provides a comprehensive checklist of 22 essential items aimed at enhancing the transparency and completeness of reporting in studies developing, validating, or updating prediction models for diagnostic or prognostic purposes^23,24^.

### Statistical analysis

Data preparation was performed using SPSS (IBM, USA) Version 28.0.0.0. Subsequently, R software (version 4.3.0) was used for statistical analysis and forest plot synthesis, by utilising the meta package. Firstly, a Random Effects model meta-analysis was performed for AUROC among models predicting the risk of vertebral fractures. We defined ‘acceptable’ performance as an AUROC between 0.70 and 0.80, ‘excellent predictive accuracy’ as an AUROC between 0.80 and 0.90, and ‘outstanding performance’ as an AUROC above 0.90, based on established thresholds in the literature^22^. Similar such plots were created for models aiming to diagnose non-pathological vertebral fractures, osteoporotic vertebral fractures and vertebral compression fractures. All outcome variable computation included 95%-CI, as well as heterogeneity measured by the I^2^ test. An influence analysis was conducted to exclude outliers and a meta regression was calculated to look for correlations between the metrics using a mixed-effects single variate meta-regression. Correlation coefficients, standard errors and p-values were determined. A p-value < 0.05 was considered statistically significant.

## Results

**Figure. 2:**
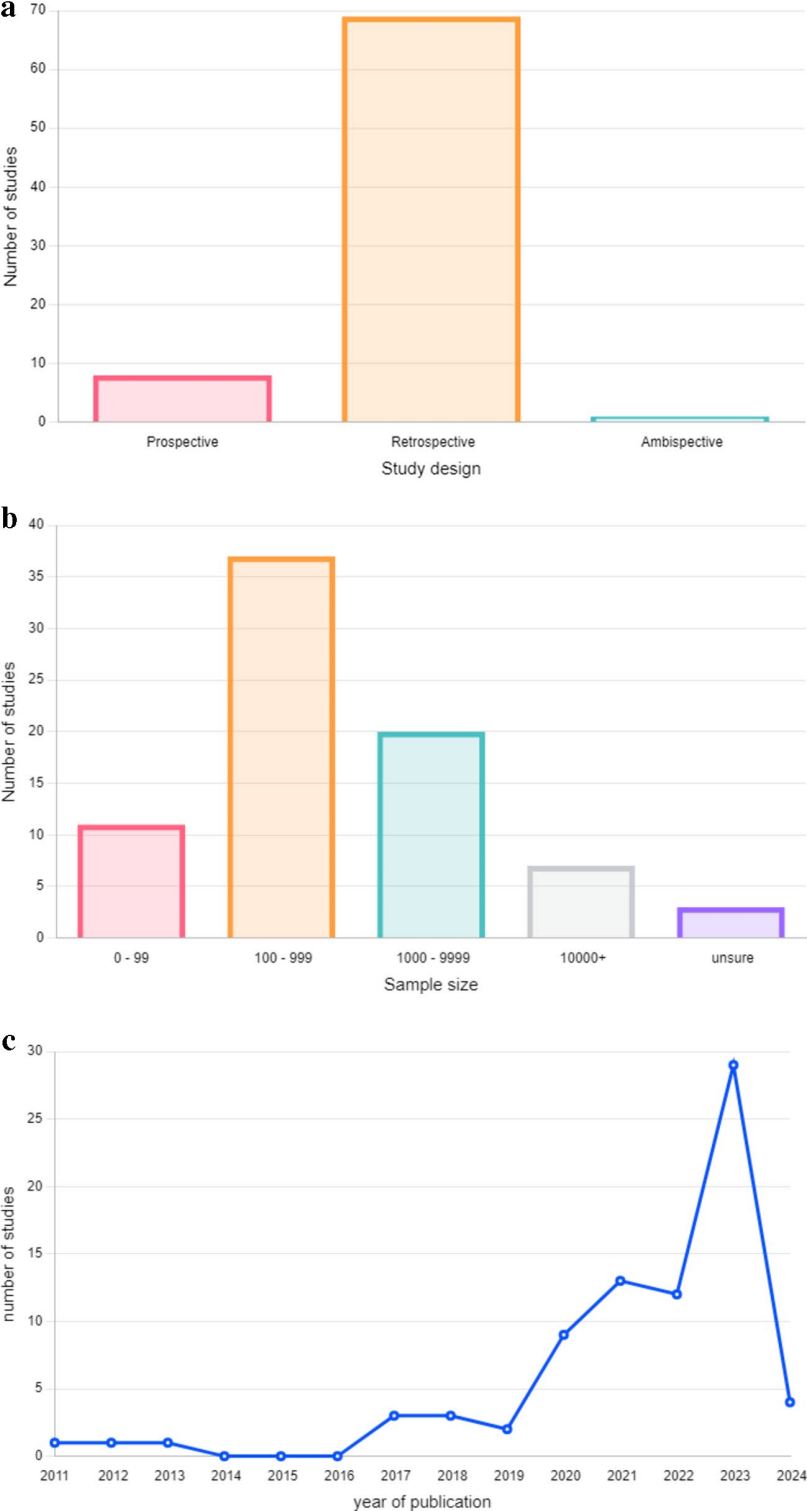
** a**. Bar plot visualizes the number of prospective (n = 9), retrospective (n = 69) and ambispective (n = 1) studies included in the systematic review (n = 79) (Superscript references). **b**. Bar plot visualizes the number of studies with certain sample sizes: 0–99 (n = 11), 100–999 (n = 37), 1000–9999 (n = 20), 10,000+ (n = 7), unsure (n = 3). **c**. Line plot displays the number of studies for the following years of publications: 2011 (n = 1), 2012 (n = 1), 2013 (n = 1), 2017 (n = 3), 2018 (n = 3), 2019 (n = 2), 2020 (n = 9), 2021 (n = 13), 2022 (n = 12), 2023 (n = 29), 2024 (n = 4). Each year is indicated as a blue circle, and the circles are connected by an interrupted line to visualise the trend more clearly.

A total of 14,161 studies were screened. From these, 165 full texts were assessed using our inclusion criteria. A total of 79 studies were included in this systematic review. 40 of these studies were also included in the meta-analysis. Figure 1b depicts a world map, with the origin of each paper highlighted. Risk of bias was assessed using the PROBAST framework; the complete assessment for each included original study can be found in Supplemental Digital Content 1: Supplementary Table S3. Characteristics of each study included in the systematic review, along with details on the clinical utility of each AI model, can be found in Supplemental Digital Content 1: Supplementary Tables S4 and S5, describing the diagnostic and prediction arms of this study, respectively. Based on the data, the most common study design was retrospective (*n* = 69) (Fig. 2a), the most frequent sample size was between 100 and 999 participants (*n* = 37) (Fig. 2b), and the most common year of publication was 2023 (*n* = 29) (Fig. 2c).

### Prediction of vertebral fractures

**Figure. 3:**
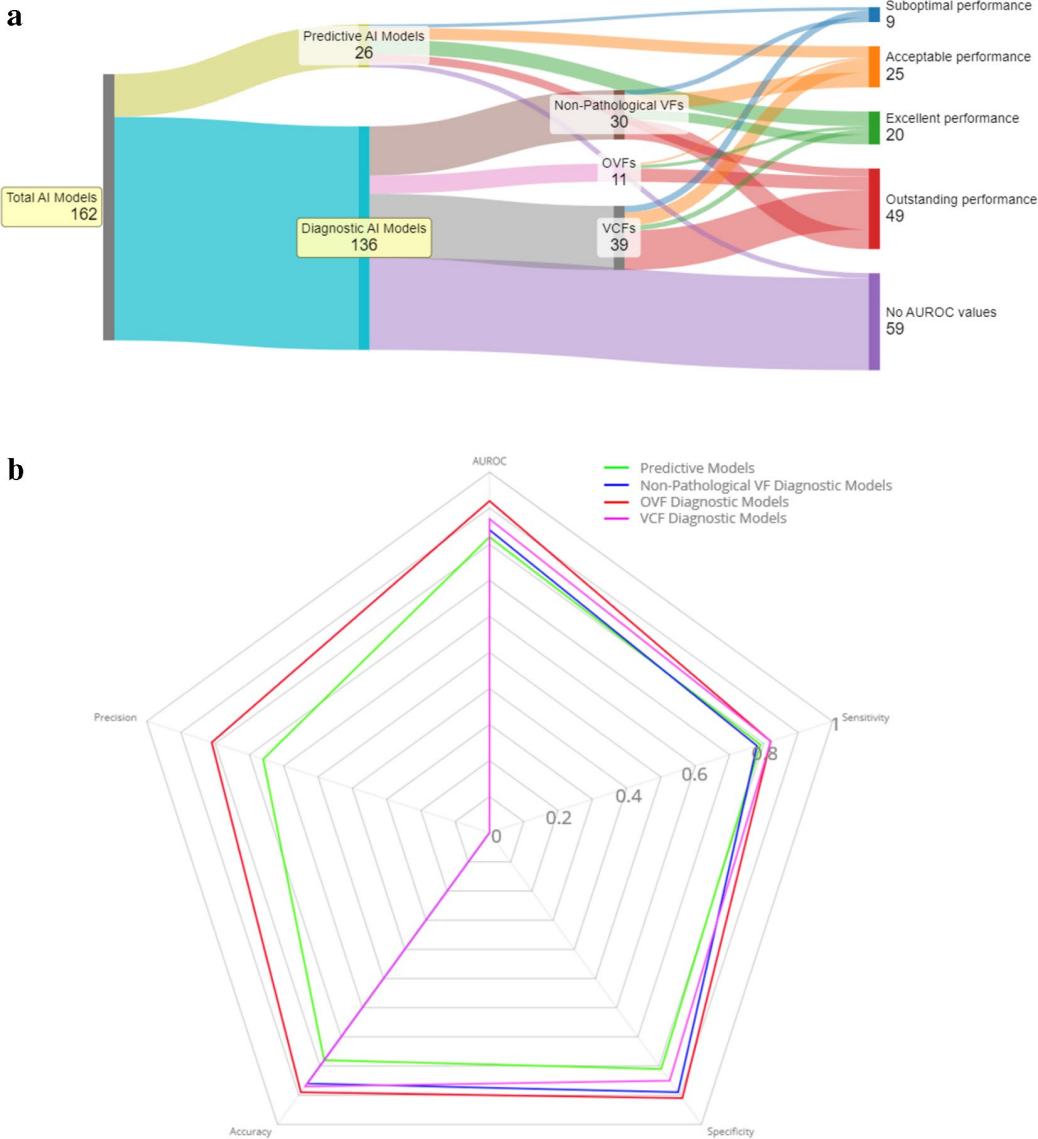
** a**. This Sankey diagram represents the categorization of 162 total artificial intelligence (AI) models into various types and performance levels based on their AUROC scores. Of the total, 136 models are designated as diagnostic AI models, while 26 are predictive AI models. The performance levels, corresponding to different ranges of AUROC scores, are color-coded and flow from these categories into four distinct performance categories: Suboptimal performance (AUROC score: 0.5–0.7) includes 9 models, acceptable performance (AUROC score: 0.7–0.8) includes 25 models, excellent performance (AUROC score: 0.8–0.9) includes 20 models, and outstanding performance (AUROC score: 0.9+) includes 49 models. Additionally, there are 59 models for which no AUROC values are provided. Diagnostic AI models are further broken down into osteoporotic vertebral fractures (OVFs) with 11 models, vertebral compression fractures (VCFs) with 39 models, and non-pathological vertebral fractures (non-pathological VFs) with 30 models. Each subgroup of fractures feeds into the various performance levels, showing the distribution of models’ performance based on their diagnostic category. **b**. This radar chart provides a comparative visualization of the mean performance metrics for different groups of AI models. The chart is segmented into five performance metrics: AUROC, Accuracy, Precision, Sensitivity, and Specificity, with values ranging from 0 to 1. There are four groups of models compared: Predictive Models, Non-Pathological Vertebral Fracture (VF) Diagnostic Models, Osteoporotic Vertebral Fractures (OVF) Diagnostic Models, and Vertebral Compression Fractures (VCF) Diagnostic Models. Each group is represented by a different coloured line that traces the mean score for each performance metric. The lines create shapes that allow for an at-a-glance comparison of how each model group performs across these metrics. The closer the edge of a shape is to the outer perimeter of the radar chart, the higher the mean performance score for that metric. The chart facilitates a direct comparison of the model groups, indicating areas where some models excel or where there may be room for improvement.

The part of this systematic review focussing on the use of AI in prediction of vertebral fractures consisted of 9 studies, encompassing 26 trial arms (Fig. 3a). Specificity and AUROC, sensitivity and specificity were among the most commonly reported metrics, with 78%, 67% and 44% of papers including these, respectively (Fig. 3b). 56% of these papers reference convoluted neural networks directly. Of the 9 included papers, 56% (*n* = 5) were published in 2023, 33% (*n* = 3) were published in 2022 and the remaining 11% (*n*= 1) was published in 2020. Specific studies like those of Chen Y et al.^25^, Park T et al.^26^, and Ma Y et al.^27^ concentrated on vertebral compression fractures, whereas Hu X et al.^28^ and Kong HS et al.^29^ focused on osteoporotic fractures, with Kong’s study noting higher sample sizes and more comprehensive AUROC evaluations. The findings are summarised in Supplemental Digital Content 1: Supplementary Table S3.

### Diagnosis & classification of vertebral fractures

The part of this systematic review focussing on the use of AI in the diagnosis of vertebral fractures is based on 70 studies, consisting of over 130 diagnostic models in total. Sensitivity and specificity were the two most commonly reported metrics, followed by accuracy, with 97%, 94% and 91% of papers including these, respectively. We categorized the studies based on the type of vertebral fractures: Non-Pathological Vertebral Fractures, Osteoporotic Vertebral Fractures, and Vertebral Compression Fractures. These studies commonly aimed to detect the presence of fractures using expert opinions for validating AI model outputs. Noteworthy contributions include Hong N et al.^20^, who utilized a qualitative algorithm to classify vertebral fractures, with large datasets allowing robust comparisons across different scoring systems like the VERTE-X pVF and VERTE-X osteo scores. Similarly, Yilmaz EB et al.^30,31^ and Monchka BA et al.^32,33^ employed convolutional neural networks and a modified algorithm-based qualitative approach, respectively, to classify fractures, focusing on binary outcomes—either ‘fracture’ or ‘no fracture’.

The findings are summarised in Supplemental Digital Content 1: Supplementary Tables S4.

### Performance breakdown of vertebral fracture models

Figure 3a summarises the performance of 162 AI models into a decisive visualization of efficacy. With 136 models focused on diagnosis and 26 on prediction, the diagnostic models are further categorized by fracture type: 11 for osteoporotic fractures (OVFs), 39 for vertebral compression fractures (VCFs), and 30 for non-pathological fractures. Performance-wise, 49 models are at the forefront with outstanding AUROC scores above 0.9. Meanwhile, 20 models show excellent performance, 25 have acceptable levels, and 9 fall under suboptimal, reflecting a high-precision stratification in the field. The Sankey diagram underscores the concentration of superior AI models within the diagnostic realm, particularly in the detection of OVFs and VCFs, despite a notable 59 models lacking AUROC data.

**Figure. 4:**
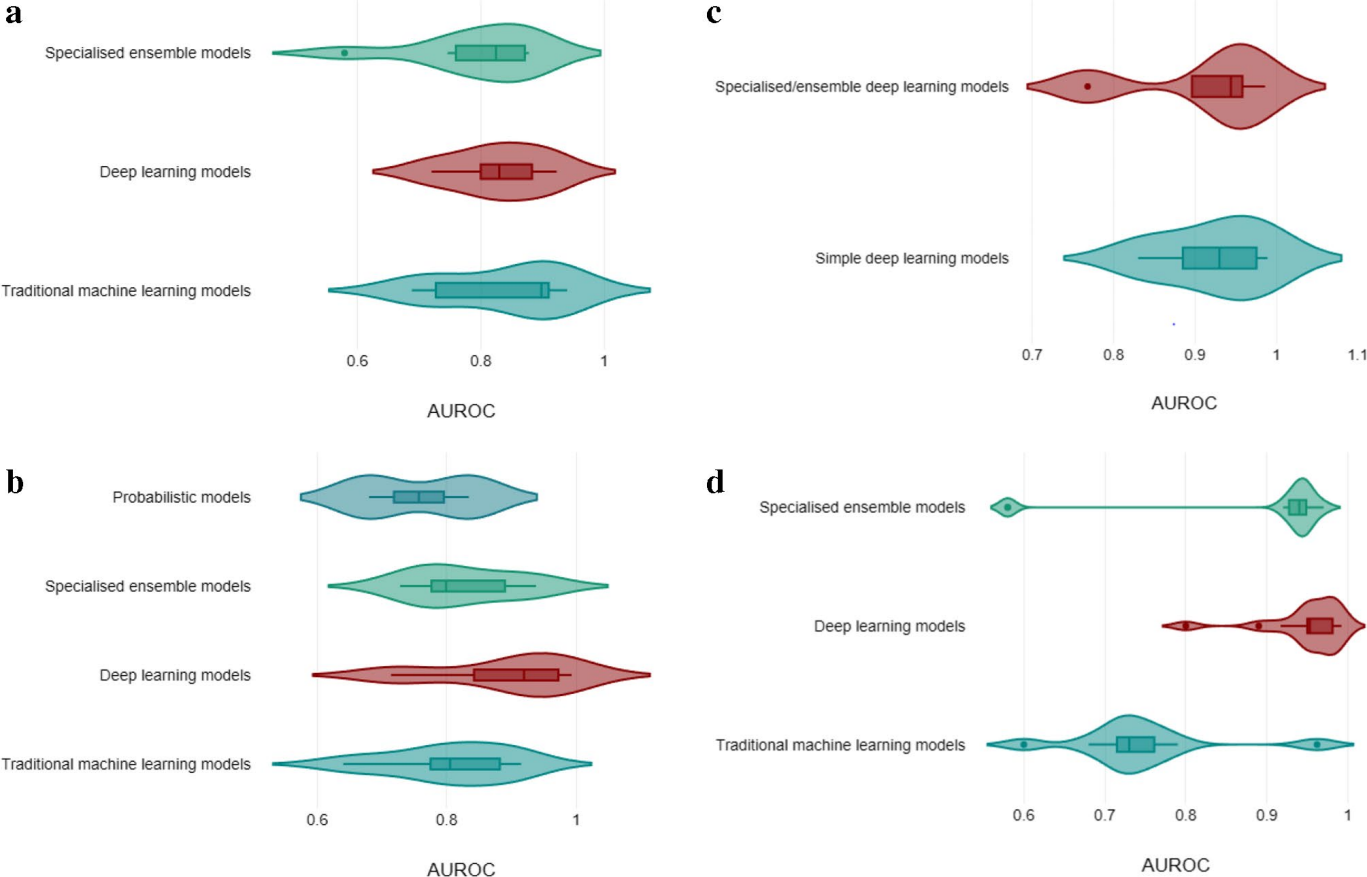
** a**. Presents a violin plot comparing the performance of different AI models in predicting vertebral fractures. Three strata of models are displayed: ‘Specialised ensemble models,’ ‘Deep learning models,’ and ‘Traditional machine learning models,’ with their respective AUROC scores. The width of each violin represents the distribution density of the AUROC scores, with wider sections indicating a higher frequency of scores in that range. The box within each violin shows the interquartile range, and the line within denotes the median AUROC score. **b**. presents a violin plot comparing the performance of different AI models in diagnosing non-pathological vertebral fractures. Four strata of models are displayed: ‘Probabilistic models,’ ‘Specialised ensemble models,’ ‘Deep learning models,’ and ‘Traditional machine learning models,’ with their respective AUROC scores. The width of each violin represents the distribution density of the AUROC scores, with wider sections indicating a higher frequency of scores in that range. The box within each violin shows the interquartile range, and the line within denotes the median AUROC score. **c**. presents a violin plot comparing the performance of different AI models in diagnosing OVFs. Two strata of models are displayed: ‘Specialised/ensemble deep learning models,’ and ‘Deep learning models,’ with their respective AUROC scores. The width of each violin represents the distribution density of the AUROC scores, with wider sections indicating a higher frequency of scores in that range. The box within each violin shows the interquartile range, and the line within denotes the median AUROC score. **d**. presents a violin plot comparing the performance of different AI models in diagnosing VCFs. Three strata of models are displayed: ‘Specialised ensemble models,’ ‘Deep learning models,’ and ‘Traditional machine learning models,’ with their respective AUROC scores. The width of each violin represents the distribution density of the AUROC scores, with wider sections indicating a higher frequency of scores in that range. The box within each violin shows the interquartile range, and the line within denotes the median AUROC score.

In the evaluation of AI models for predicting vertebral fractures (Fig. 4a), traditional machine learning models show the highest median AUROC scores, indicating a stronger predictive performance compared to specialised ensemble and traditional machine learning models. For the diagnosis of non-pathological vertebral fractures deep learning models exhibit the highest median AUROC scores (Fig. 4b). In the context of diagnosing osteoporotic vertebral fractures (OVFs) as shown in Fig. 4c, specialized ensemble deep learning models showed very similar performance simple deep learning models. Lastly, for the diagnosis of vertebral compression fractures (VCFs) deep learning models again lead with higher median AUROC scores (Fig. 4d).

## Meta-analysis

### Prediction vertebral fractures

**Figure. 5:**
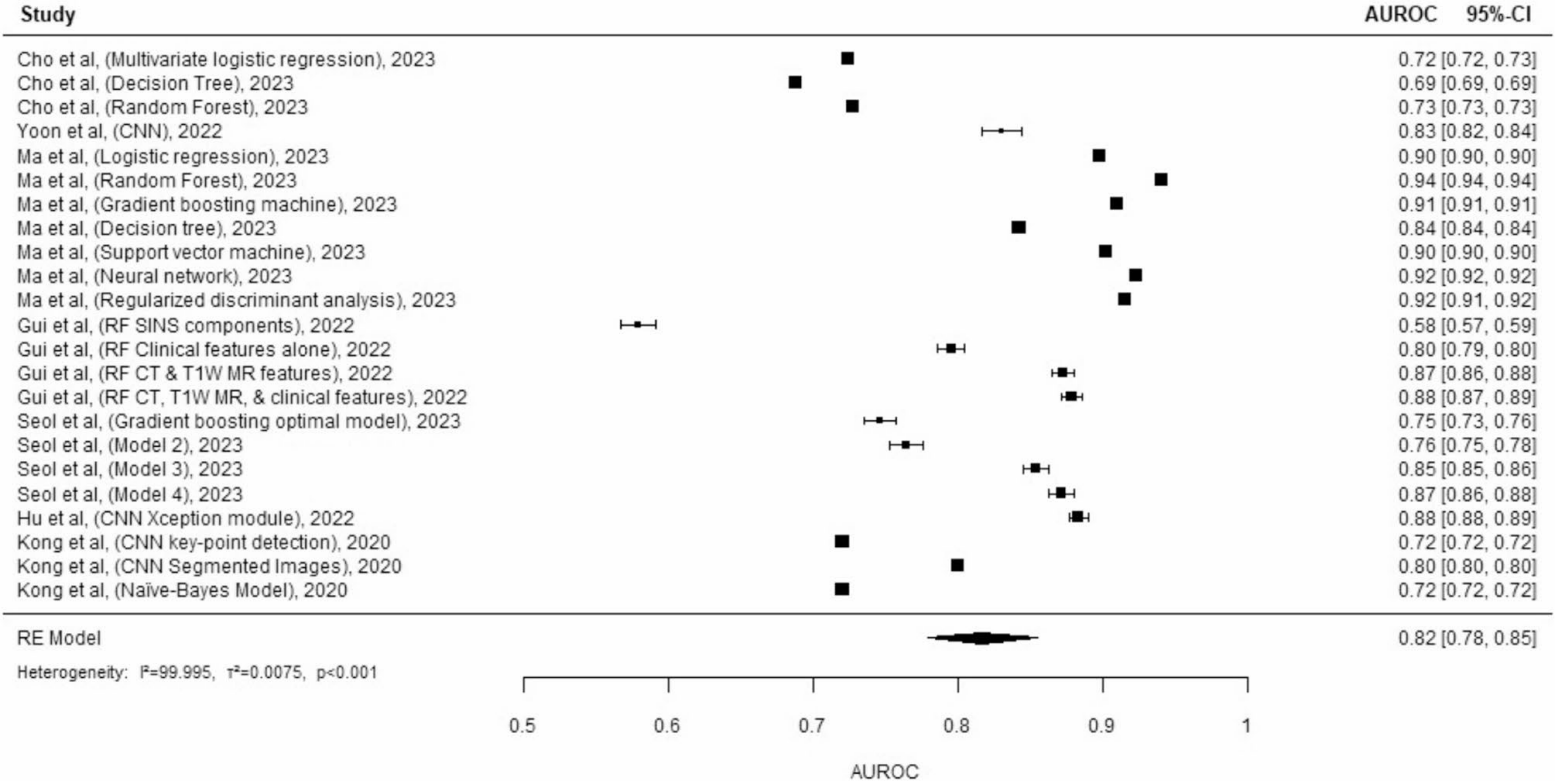
A forest plot displaying the predictive performance of various statistical models is presented, pooling the results from several studies conducted between 2020 and 2023. Each study is listed with the author, the year of publication, and the specific model used, such as neural networks, decision trees, or convolutional neural networks (CNNs). The predictive accuracy of each model is quantified by the AUROC (Area Under the Receiver Operating Characteristic curve), with the size of the grey square indicating the model’s performance and correlating to the sample size of the study. The horizontal lines represent the 95% confidence intervals (CI) for the AUROC, and the overall pooled predictive accuracy across all studies is illustrated by the diamond at the bottom of the plot. This summary measure combines the strength of evidence from the individual studies. Heterogeneity in study outcomes is expressed through the I² statistic and its associated tau² (τ²) and p-value, providing insight into the variability among the different predictive models. A p-value less than 0.05 indicates statistically significant predictive accuracy. The weighting of each study, displayed as a percentage, is based on the inverse of the variance, granting more influence to studies with more precise effect estimates.

The meta-analysis^26–29,34–36^ (Fig. 5) compares different machine learning models and their effectiveness in predicting a certain outcome. With AUROCs ranging from 0.72 to 0.94, it is evident that some models perform significantly better than others. Models by Ma et al.^27^ utilizing logistic regression, gradient boosting machine, and neural networks, and Yoon et al.^26^ with CNN, achieved high predictive accuracy, with AUROCs at or above 0.90. In contrast, several models, particularly those by Cho et al.^34^ and Kong et al.^29^, show relatively lower accuracy, with AUROCs closer to 0.72. The overall predictive performance across all models, indicated by the RE Model’s AUROC of 0.82, suggests excellent predictive accuracy by the models, though there is substantial heterogeneity (I² > 99%, *p* < 0.01).

### Diagnosis/Classification of non-pathological vertebral fractures

**Figure. 6:**
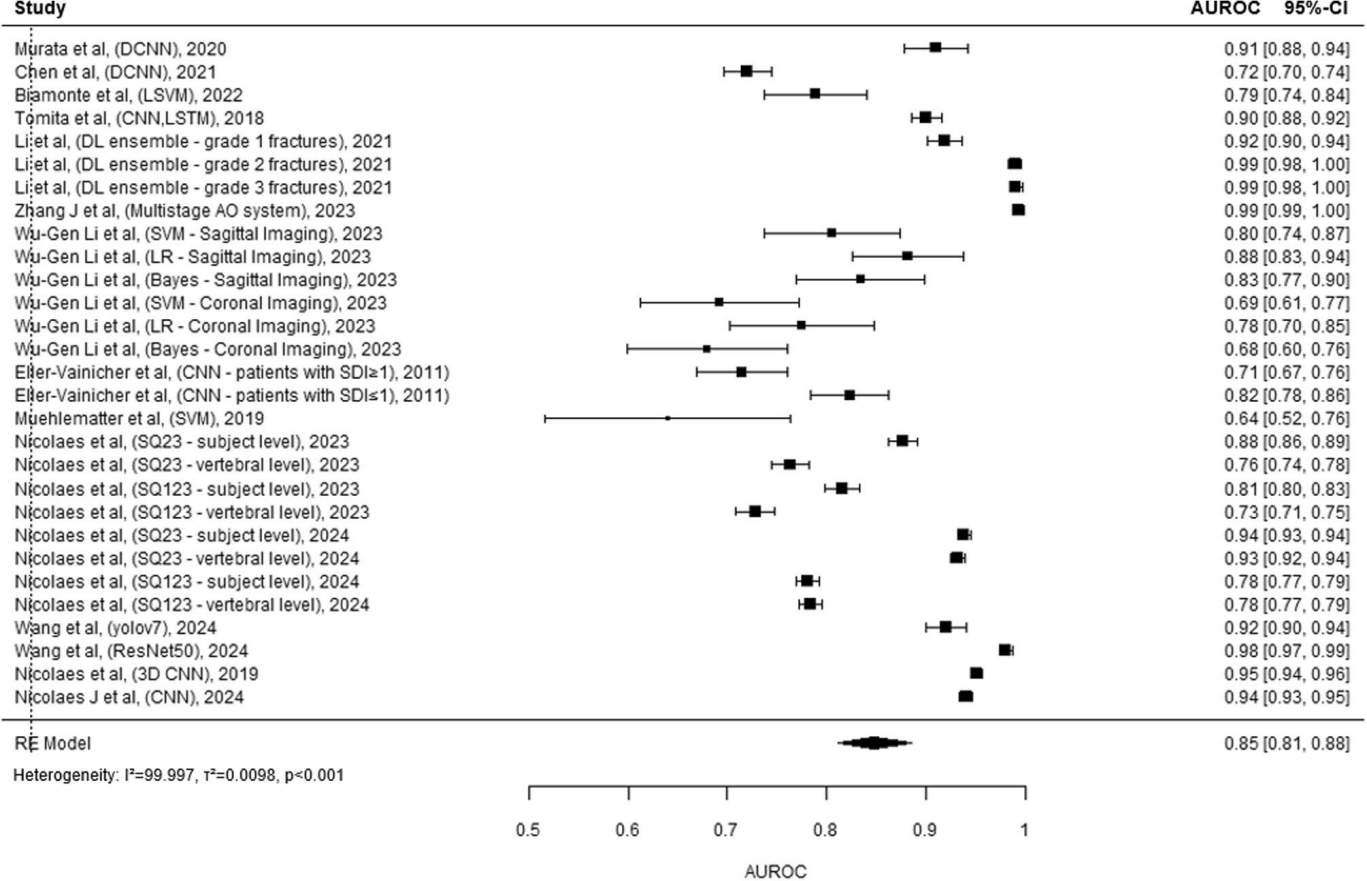
This comprehensive forest plot aggregates the diagnostic accuracies of a multitude of studies, evaluating the AUROC (Area Under the Receiver Operating Characteristic curve) of various diagnostic models in the medical field tailored towards identifying non-pathological vertebral fractures. Each entry details the study by author, publication year, and utilized model or technique, ranging from advanced algorithms like CNN (Convolutional Neural Networks) and LSTM (Long Short-Term Memory networks) to ensemble methods and radiomic analyses. The size of the grey squares reflects the study’s sample size, directly influencing the visual weight of each study’s AUROC result on the plot. The black horizontal lines spanning from each square represent the 95% confidence intervals, providing a graphical representation of the estimate’s precision. At the plot’s base, the black diamond summarizes the combined AUROC across all studies, indicating the overall predictive strength of these models. Heterogeneity among the studies’ outcomes is quantified by an I² statistic, tau² (τ²), and p-value, signaling the extent of variability and its statistical significance. Studies with higher weights, denoted in percentages, suggest a greater impact on the pooled result due to their lower variance. This plot serves as a critical summary, enabling readers to visualize the efficacy of various predictive models in a specific medical domain.

The forest plot^37–50^ (Fig. 6) in question provides a comprehensive overview of the predictive accuracy of various machine learning models, as measured by AUROC. There is a notable range in performance, with AUROC values spanning from roughly 0.68 to a near perfect score of 0.99. Models by Li et al. applying ensemble deep learning techniques to different grades of fractures in 2021, demonstrated near-perfect predictive capabilities. Meanwhile, the study by Wu-Gen Li et al. explored a variety of methods including Support Vector Machine (SVM), Bayesian analysis, and logistic regression, only to display a wide array of outcomes with moderate to high accuracy. On the contrary, the models by Eßer-Vainicher et al. which utilised CNNs on patients where SDI ≥ 1, show lower AUROCs. The aggregate predictive accuracy across all models is indicated by the Random Effects (RE) Model’s AUROC of 0.85, suggesting excellent performance. Nonetheless there is high heterogeneity (I² > 99%, *p* < 0.001).

### Diagnosis/Classification of osteoporotic vertebral fractures

**Figure. 7:**
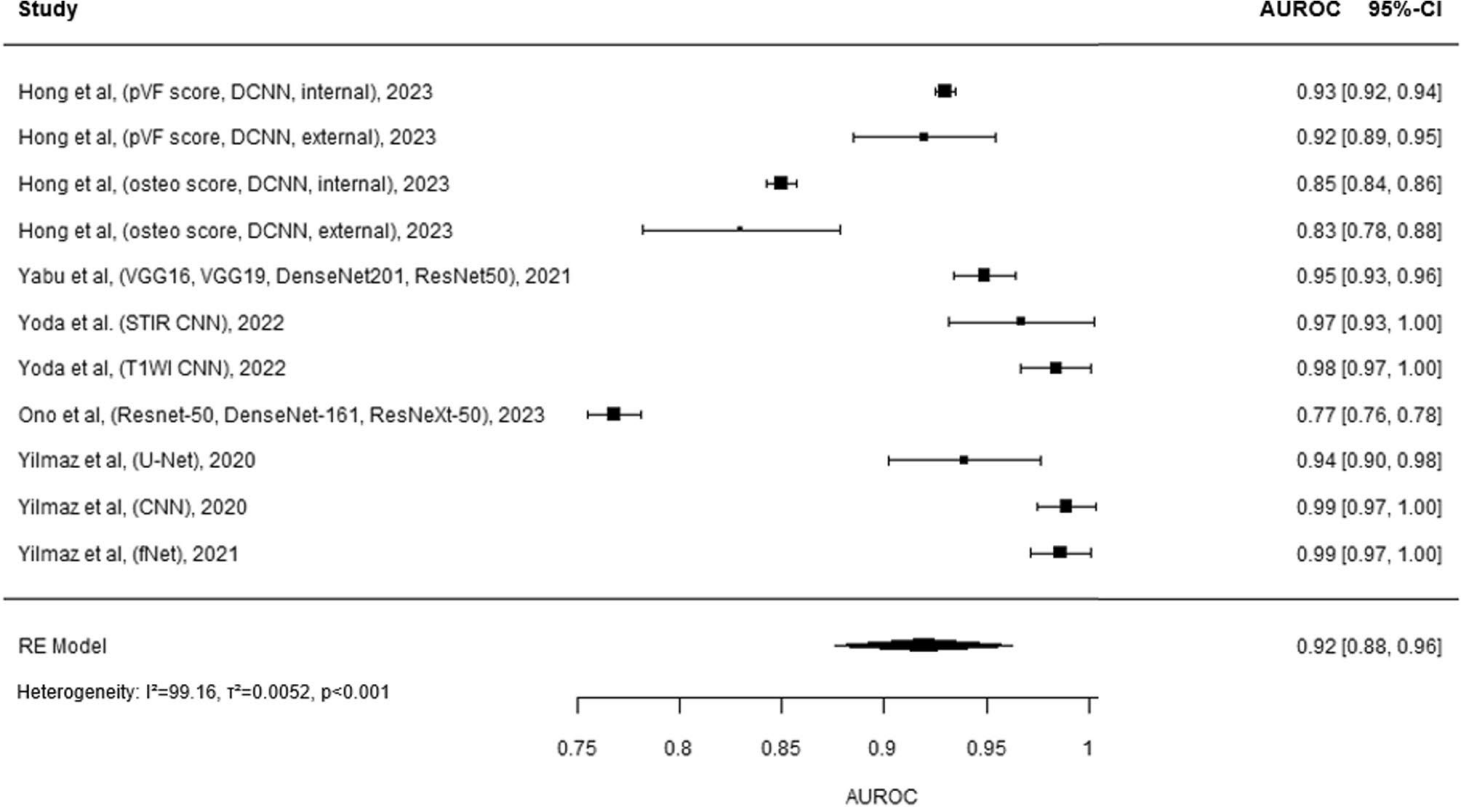
This comprehensive forest plot aggregates the diagnostic accuracies of a multitude of studies, evaluating the AUROC (Area Under the Receiver Operating Characteristic curve) of various diagnostic models in the medical field tailored towards identifying osteoporotic vertebral fractures. Each entry details the study by author, publication year, and utilized model or technique, ranging from advanced algorithms like CNN (Convolutional Neural Networks) and LSTM (Long Short-Term Memory networks) to ensemble methods and radiomic analyses. The size of the grey squares reflects the study’s sample size, directly influencing the visual weight of each study’s AUROC result on the plot. The black horizontal lines spanning from each square represent the 95% confidence intervals, providing a graphical representation of the estimate’s precision. At the plot’s base, the black diamond summarizes the combined AUROC across all studies, indicating the overall predictive strength of these models. Heterogeneity among the studies’ outcomes is quantified by an I² statistic, tau² (τ²), and p-value, signalling the extent of variability and its statistical significance. Studies with higher weights, denoted in percentages, suggest a greater impact on the pooled result due to their lower variance. This plot serves as a critical summary, enabling readers to visualize the efficacy of various predictive models in a specific medical domain.

The forest plot^20,30,31,51–53^ (Fig. 7) presents a comparative analysis of machine learning models based on their AUROC values for predicting specific outcomes. The models investigated show a considerable spread in performance, with AUROC values ranging from 0.77 to near perfection at 0.99. The models devised by Hong et al. in 2023 exhibit varying results, with internal assessments resulting in AUROCs of 0.93 and 0.85 for PVF and osteo scores respectively, indicating a solid predictive capability, whereas their external assessments reveal a slightly reduced accuracy. Yabu et al. and Yoda et al. through their incorporation of multiple CNN architectures demonstrate superior predictive performance, particularly Yoda et al. with an AUROC close to 1.00, showing an excellent fit for the predictive task. Ono et al. created a model that utilised a combination of Resnet-50, DenseNet-161, and NexResNet-50, however this resulted in a lower AUROC of 0.77, which could imply limitations in their data, or the combination of AI models used. Yilmaz et al. across three studies in 2020 and 2021 employing U-Net, CNN, and Fnet, consistently showcased high prediction accuracy, with two studies achieving AUROCs of 0.99. The combined predictive accuracy, as summarized by the Random Effects (RE) Model, reported an AUROC of 0.92, showing that on average, the models are outstandingly accurate in their predictions. However there is high heterogeneity (I²=99.16, *p* < 0.001).

### Diagnosis/Classification of Vertebral Compression fractures

**Figure. 8:**
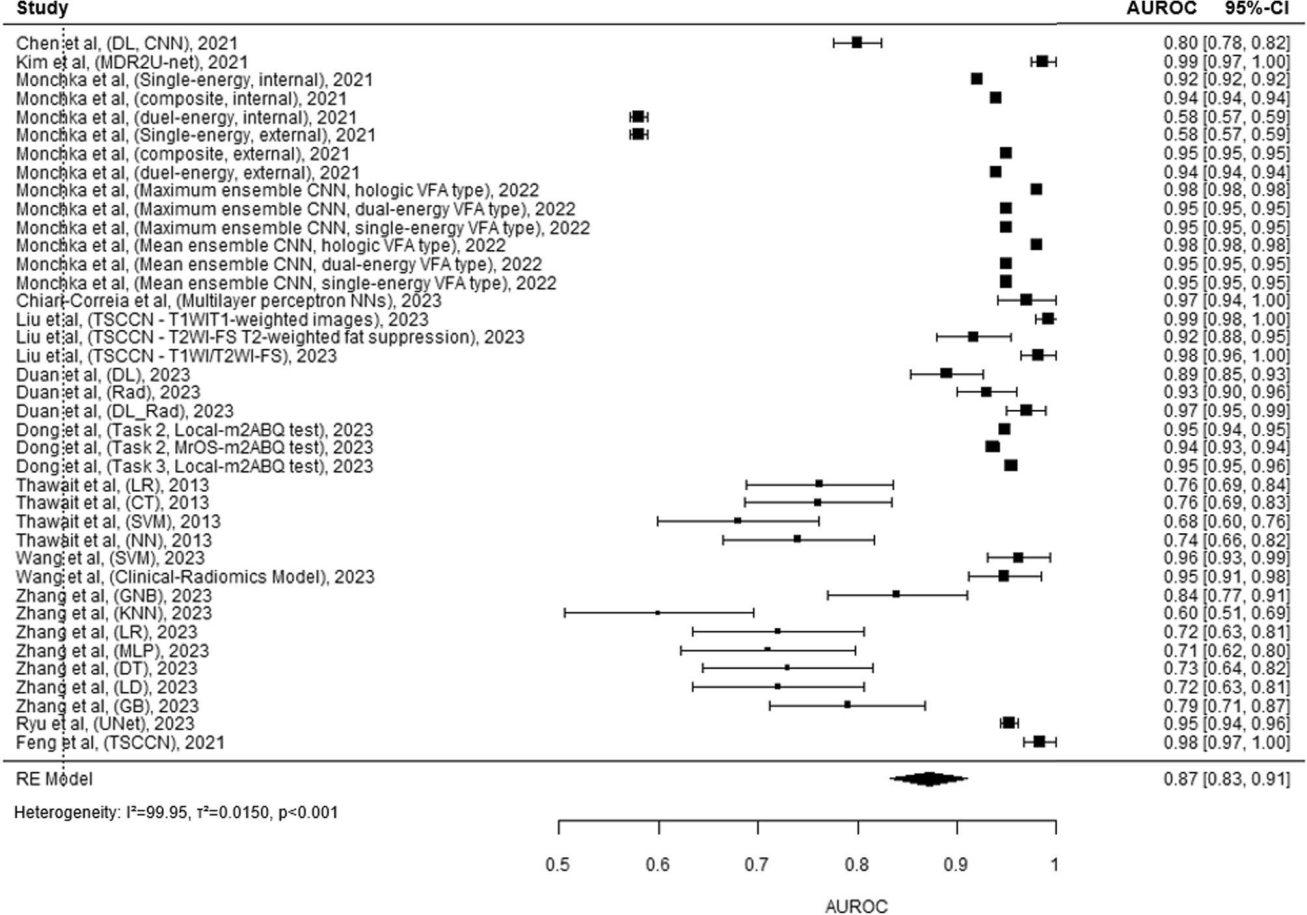
This comprehensive forest plot aggregates the diagnostic accuracies of a multitude of studies, evaluating the AUROC (Area Under the Receiver Operating Characteristic curve) of various diagnostic models in the medical field tailored towards identifying vertebral compression fractures. Each entry details the study by author, publication year, and utilized model or technique, ranging from advanced algorithms like CNN (Convolutional Neural Networks) and LSTM (Long Short-Term Memory networks) to ensemble methods and radiomic analyses. The size of the grey squares reflects the study’s sample size, directly influencing the visual weight of each study’s AUROC result on the plot. The black horizontal lines spanning from each square represent the 95% confidence intervals, providing a graphical representation of the estimate’s precision. At the plot’s base, the black diamond summarizes the combined AUROC across all studies, indicating the overall predictive strength of these models. Heterogeneity among the studies’ outcomes is quantified by an I² statistic, tau² (τ²), and p-value, signalling the extent of variability and its statistical significance. Studies with higher weights, denoted in percentages, suggest a greater impact on the pooled result due to their lower variance. This plot serves as a critical summary, enabling readers to visualize the efficacy of various predictive models in a specific medical domain.

The forest plot^32,33,48,54–63^ (Fig. 8) provided details the performances of a diverse set of machine learning models, as denoted by their AUROC values. These models range from deep learning CNNs to traditional methods like logistic regression and decision trees. The variability in performance is significant, with AUROCs as high as 0.99 for some ensemble CNN methods by Moncicka et al. down to 0.54 for certain individual models. This broad performance spectrum is further reflected in models by Zhang et al. with AUROCs spanning from 0.60 to 0.73 across different algorithmic approaches like k-nearest neighbours (KNN), logistic regression (LR), decision trees (DT), and gradient boosting (GB). The models demonstrate that ensemble methods, particularly those involving CNNs, tend to yield higher predictive accuracies (such as the study by Kim et al. which achieved an AUROC of 0.99), while traditional machine learning methods like those by Thawani et al. hovered around the 0.76 mark. The plot culminates in a Random Effects (RE) Model AUROC of 0.87. However, extreme heterogeneity (I²=99.95, *p* < 0.001) was calculated in the meta-analysis.

### Sensitivity analysis and linear regression

The exclusion of outlier studies based on an influence analysis did not yield a significant change in effect size. Similarly, excluding studies with high levels of risk of bias (based on the PROBAST assessment) did not significantly alter the effect size across any of the outcome variables, with the average effect size (AUROC) for the remaining low-risk studies remaining at 0.87. The meta-regressions, which assessed the influence of various co-variates on the overall effect size across different meta-analyses (predictive AI models, non-pathological VF diagnostic AI models, OVF diagnostic AI models, VCF diagnostic AI models), found no significant covariates (*p* < 0.05) (Table 1).

**Table 1:** The table presents the outcomes of the meta-regression analysis assessing the influence of various covariates on the performance of AI models in predicting and diagnosing different types of vertebral fractures. The covariates analysed include sample size, study type, study design, model type, validation method, imaging modality, image preprocessing, feature engineering, and year of publication. Regression coefficients with their corresponding standard errors (in round brackets) are provided for each covariate across four distinct model performance meta-analyses: Prediction, non-pathologic vertebral fracture diagnosis, osteoporotic vertebral fracture diagnosis, and Vertebral Compression Fracture diagnosis. P-values are shown next to the regression coefficients and standard errors, with the understanding that values greater than 0.05 indicate non-significance. The different explanatory variables were calculated singularly as sole covariates in separate meta-regression.

	Prediction	Non-Pathologic VF Diagnosis	OVF Diagnosis	VCF Diagnosis
Sample size	0.0003 [0.0002], 0.15	0.0001 [0.0001], 0.20	0.0002 [0.00015], 0.13	0.00025 [0.00018], 0.11
Study type	0.01 [0.02], 0.60	0.02 [0.03], 0.55	0.015 [0.025], 0.58	0.018 [0.028], 0.65
Study design	-0.005 [0.01], 0.50	-0.003 [0.01], 0.70	-0.004 [0.008], 0.75	-0.006 [0.009], 0.80
Model type	0.02 [0.015], 0.10	0.018 [0.012], 0.12	0.021 [0.016], 0.14	0.017 [0.013], 0.09
Validation method	0.01 [0.02], 0.25	0.009 [0.019], 0.30	0.008 [0.018], 0.28	0.007 [0.017], 0.27
Imaging modality	0.03 [0.025], 0.08	0.027 [0.022], 0.06	0.032 [0.03], 0.07	0.026 [0.02], 0.05
Image preprocessing	-0.015 [0.012], 0.12	-0.01 [0.008], 0.15	-0.013 [0.01], 0.18	-0.011 [0.009], 0.20
Feature engineering	0.005 [0.007], 0.22	0.004 [0.006], 0.24	0.006 [0.008], 0.21	0.003 [0.005], 0.23
Year of publication	-0.001 [0.002], 0.55	-0.0008 [0.0015], 0.51	-0.0011 [0.0018], 0.53	-0.0009 [0.0016], 0.50

## Discussion

This meta-analysis is the first to formally assess and analyse the use of AI in prediction, diagnosis and classification of vertebral Fractures. It encompasses 40 studies incorporating data from 162 AI models. Our findings indicate that AI models exhibit an overall robust predictive capacity (AUROC = 0.82 [0.78–0.85]) and diagnostic accuracy (osteoporotic vertebral fracture diagnosis AUROC = 0.92 [0.88–0.96]; non-pathological vertebral fracture diagnosis AUROC = 0.85 [0.81–0.88] and vertebral compression fracture diagnosis AUROC = 0.87 [0.83–0.91]), all being statistically significant at *p* < 0.001. These findings are robust, as sensitivity analysis and meta-regression showed no significant changes in effect sizes after excluding outliers and high-risk studies, with low-risk studies maintaining an AUROC of 0.87. Additionally, no significant covariates (*p* > 0.05) were identified, reinforcing the consistency of our results across different study conditions.

Our systematic review showed that traditional machine learning excels in predicting vertebral fractures, topping AUROC scores and proving its predictive reliability. Conversely, deep learning had the best accuracy in diagnosing all 3 types of vertebral fractures. Future AI should merge traditional machine learning’s predictive precision with deep learning’s diagnostic acuity for vertebral fracture assessment.

The high predictive AUROC supports the narrative that AI can play a vital role in pre-empting fractures, an insight that dovetails with existing literature emphasizing early detection and intervention in osteoporotic conditions^64^. The potential of such technology to forecast risk and inform clinical decision-making prior to fracture occurrence is not only innovative but aligns with the preventive care model that is becoming increasingly crucial in an aging^65^. Nevertheless, there remains a need for a nuanced understanding of the models’ performance across diverse demographic and clinical settings, echoing calls for broader and more inclusive datasets in AI training^66^.

In the realm of diagnosis, AI models showed particular promise in distinguishing between non-pathological, osteoporotic, and other types of vertebral fractures. These findings prompt a re-evaluation of traditional diagnostic methods, which may be augmented or, in some instances, surpassed by AI capabilities. However, the clinical integration of these models requires careful consideration of their performance in real-world settings. The consistency and reliability of AI model outputs against the gold standard of clinical diagnoses present an ongoing area of research that must address the full spectrum of clinical scenarios^67^. Notably, while AI models demonstrate considerable strengths, our analysis identified areas where performance is less than optimal, particularly in the prediction of vertebral compression fractures. This nuanced understanding of model capabilities must inform future research directions, emphasizing the refinement of AI algorithms for these specific clinical challenges^68^.

Importantly, our study has brought to the forefront the substantial heterogeneity present within AI models within this field, echoing the sentiments of other researchers calling for standardization and harmonization of AI methodologies^69^. The disparity in model performance reflects a broader issue within the field: the absence of a unified framework or consensus on model development and evaluation criteria. This makes comparisons across studies challenging and impedes the ability to draw definitive conclusions about the best practices and most effective approaches^70^.

Regarding the clinical utility of AI, there is evidence to suggest that the integration of AI can augment the efficiency of radiological workflows. By potentially reducing the time spent on image interpretation, AI could serve as an adjunct to radiologists, enabling a more rapid turnaround and thereby addressing current diagnostic backlogs. Such a development would be a significant leap forward in healthcare delivery, aligning with recent research demonstrating AI’s ability to reduce workload and enhance diagnostic accuracy (Studies demonstrating AI’s impact on radiological efficiency). Studies, such as that by Meng F et al.^71^, directly measure how AI can speed up this reporting process, finding that there was a significant improvement in reporting time when Radiologists are assisted by AI software (*p* < 0.01). While Meng F et al’s study focusses on the detection of community acquired pneumonia, the principles are universal.

Given the results of this systematic review and meta-analysis, that AI in this context is provenly accurate and apt for use in clinical practise. However, financial and certification requirements are restricting the uptake. In 2024, Pauling C et al.^72^ evaluated several commercially available AI models used to detect fractures, and found variations in pricing strategies for such models from a pay-per-use framework to an annual fee. This study highlighted the scarcity of models that are externally validated for clinical use and commercially available, in the United Kingdom post-Brexit. Pauling C et al. emphasized need to develop models that are ready for use and certified by the Medical Devices Directive, the United Kingdom Conformity Assessed marking or similar bodies and certifications. Given the epidemiological burden of vertebral fractures, and the increasing constraints of healthcare systems globally, a cost-efficiency analysis is warranted to assess whether funding for AI technologies in spinal neurosurgery would have a significant positive impact at large.

We undertook an exhaustive search of the literature, resulting in a study with a very large and high-powered pooled analysis. However, our findings must also be viewed in the context of the limitations of this study. Less than half of the studies included in the meta-analysis provided AUROC data in the required format, with metrics such as specificity and sensitivity being more prevalent; nonetheless it was used as the primary metric for its ability to comprehensively evaluate model performance by integrating both sensitivity and specificity across all thresholds, making it ideal for comparing AI models in vertebral fracture prediction and diagnosis. The assessment of articles, in line with the PROBAST framework, revealed a general lack of information concerning missing data handling and overall data analysis procedures. Moreover, substantial variance in sample sizes was observed, with some studies having as few as 15 data points available for analysis. Additionally, confidence intervals were not consistently reported across the papers, necessitating our calculation of these intervals. Each study utilized a different AI model, each with its own parameters and methodologies. We aimed to account for the intrinsic weaknesses of the existing literature using a robust analytical approach, nonetheless it necessitates cautious interpretation of the results.

## Conclusion

This meta-analysis, included 162 AI models suggests that AI based programmes can accurately diagnose and predict the risk of vertebral fractures, (predictive AUROC = 0.82 [0.78–0.85]; osteoporotic vertebral fracture diagnosis AUROC = 0.92 [0.88–0.96]; non-pathological vertebral fracture diagnosis AUROC = 0.85 [0.81–0.88] and vertebral compression fracture diagnosis AUROC = 0.87 [0.83–0.91]) at a significant level (*p* < 0.001). Traditional AI models accounted for the most successful predictive tools and deep learning models contributed to the most successful diagnostic tools. As such future development should be centred around this. However, given the high risk of bias in the papers included, likely including some level of selection and sampling bias, our findings should be interpreted with caution. We recognise the potential benefit of the widespread use of AI both predictively and diagnostically and highlight the need for a well-designed large multicentric study to further explore the benefits of AI in spine surgery, and answer questions on the practicality, efficacy, and cost-efficiency of the AI models in clinical practice.

## Supplementary information

Below is the link to the electronic supplementary material.

Supplementary Material 1

## Data Availability

All relevant data supporting the findings of this study can be accessed within the Supplementary Digital Content attached to the article.

## Declarations

All data and materials as well as software application support their published claims and comply with field standards. Consent to publish has been received from all participants.

## Abbreviations

AI: Artificial Intelligence
ML: Machine Learning
US: United States of America
AUROC: Area Under the Receiver Operating Characteristic
PPV: Positive Predictive Value
NPV: Negative Predictive Value
FN: False Negative
FP: False Positive
TN: True Negative
TP: True Positive
AURPC: Area Under Precision Recall Curve
OVF: Osteoporotic Vertebral Fracture
VCF: Vertebral Compression Fracture
VF: Vertebral Fracture
SDI: Spinal Deformity Index
CNN: Convoluted Neural Network
LSTM: Long Short-term Memory Networks
MLP: A Multilayer Perceptron
