# Supplementary material for: Artificial intelligence in risk prediction and diagnosis of vertebral fractures
Source: Sci Rep. 2024 Dec 19;14:30560. doi: 10.1038/s41598-024-75628-2 (PMC11659610; doi:10.1038/s41598-024-75628-2)
Supplement: Supplementary file 1 — Supplementary Material 1 [file 41598_2024_75628_MOESM1_ESM.docx]

**Supplemental Digital Content 1**

**Artificial Intelligence and Machine Learning**

**for Risk Prediction and Diagnosis of Vertebral Fractures: A Systematic Review and Meta-analysis**

Srikar R. Namireddy^1,2^ ;

Saran S Gill^1.2^ ; Amaan Peerbhai^1,2^ ; Abith G Kamath^1,2^

Daniele S. C. Ramsay^1,2^ ; Hariharan Subbiah Ponniah^1,2^ ;

Dragan Jankovic^3^ ; Darius Kalasauskas^3^ ; Jonathan Neuhoff^4^ ;

Andreas Kramer^3^ ; Salvatore Russo^5^

Santhosh G. Thavarajasingam^1,3 *^

**INSTITUTION:**

1. Imperial Brain & Spine Initiative, Imperial College London, London, United Kingdom
2. Faculty of Medicine, Imperial College London, London, United Kingdom
3. Department of Neurosurgery, University Medical Center Mainz, Mainz, Germany
4. Center for Spinal Surgery and Neurotraumatology, Berufsgenossenschaftliche Unfallklinik Frankfurt am Main, Germany
5. Department of Neurosurgery, Imperial College Healthcare NHS Trust, London

Table of Contents

[Supplementary Material 1: 3](#_Toc138678524)

[Supplementary Table 1: 5](#_Toc138678524)

[Supplementary Table 2: 8](#_Toc138678525)

[Supplementary Table 3 9](#_Toc138678526)

[Supplementary Table 4 21](#_Toc138678526)

[Supplementary Table 4 25](#_Toc138678527)

[References 51](#_Toc138678550)

**Supplementary Material 1:** The study protocol used for this review.

### **Review Methods**

- **Review question:** Is artificial intelligence (AI) effective in diagnosing and predicting vertebral fractures?
- **Searches:** Searches were conducted across MEDLINE, Embase, Scopus, PubMed, and Web of Science Library. The search string is available in Supplemental Digital Content 1: Supplementary Table S1.
- **Condition or domain being studied:** Vertebral fractures, including non-pathological, osteoporotic, and vertebral compression fractures.
- **Participants/population:** Studies including human subjects with vertebral fractures, aged 18 years and older.
- **Intervention(s), exposure(s):** Use of AI, including machine learning models, for diagnosing or predicting vertebral fractures.
- **Comparator(s)/control:** Studies comparing AI models with traditional diagnostic methods or models without AI.
- **Types of study to be included:** Prospective, retrospective, and ambispective studies that report on AI in the context of vertebral fractures.
- **Context:** Studies from various clinical settings, including hospitals and research centers.

### **Eligibility Criteria**

- **Inclusion criteria:** Peer-reviewed studies published in English, involving adult human patients with vertebral fractures, utilizing AI/ML models for diagnosis or prediction of fractures.
- **Exclusion criteria:** Case reports, letters to the editor, narrative reviews, books, studies not evaluating AI performance, non-English studies, studies focusing on non-vertebral fractures.
- **Language restrictions:** English only.
- **Publication status:** Only peer-reviewed, published articles were included.

### **Data Extraction and Risk of Bias Assessment**

- **Data extraction (selection and coding):** Data was extracted using COVIDENCE for duplicate removal and title and abstract screening. Data included study characteristics, AI model details, performance metrics (AUROC, sensitivity, specificity), and risk of bias assessments.
- **Risk of bias (quality) assessment:** The PROBAST tool was used to assess the risk of bias, examining participants, predictors, outcomes, and analysis.
- **Strategy for data synthesis:** A Random Effects model meta-analysis was conducted, focusing on AUROC as the primary metric for model performance. Forest plots and influence analyses were performed.
- **Assessment of heterogeneity:** Heterogeneity was assessed using the I² statistic.
- **Subgroup analysis:** Subgroup analyses were conducted based on the type of vertebral fracture (non-pathological, osteoporotic, vertebral compression).
- **Sensitivity analysis:** Sensitivity analyses were conducted to exclude outliers and high-risk studies.

**Supplementary Table 1:** The search strategy performed on February 12th, 2024, is shown below outlining the respective databases, the search terms, and number of results from each database.

| **Database** | **Search terms** | **Results (n)** |
| --- | --- | --- |
| Embase | (("Vertebral    Compression Fractures" or "Spinal Fractures" or    "Compression Fractures" or "Vertebral" or "Vertebral    Fracture*" or ("Vertebral" and "Compression" and    "Fracture*OR Trauma")) and ("Algorithms" or    "Artificial Intelligence" or "AI" or "Machine    Learning" or "Neural Networks, Computer" or "Random    Forest" or "artificial neural network" or "ANN" or    "Support Vector Machine" or "support vector machine" or    "SVM" or "random forest" or "gradient boosting"    or "Decision Trees" or "decision tree" or    "convolutional neural network" or "CNN" or    "Algorithm*" or "Computer Vision" or    ("Artificial" and "Intelligence"))).mp. [mp=title, book    title, abstract, original title, name of substance word, subject heading    word, floating sub-heading word, keyword heading word, organism supplementary    concept word, protocol supplementary concept word, rare disease supplementary    concept word, unique identifier, synonyms, population supplementary concept    word, anatomy supplementary concept word] | N = 2871 |
| MEDLINE | (("Vertebral Compression Fractures" or "Spinal Fractures" or "Compression Fractures" or "Vertebral" or "Vertebral Fracture*" or ("Vertebral" and "Compression" and "Fracture*OR Trauma")) and ("Algorithms" or "Artificial Intelligence" or "AI" or "Machine Learning" or "Neural Networks, Computer" or "Random Forest" or "artificial neural network" or "ANN" or "Support Vector Machine" or "support vector machine" or "SVM" or "random forest" or "gradient boosting" or "Decision Trees" or "decision tree" or "convolutional neural network" or "CNN" or "Algorithm*" or "Computer Vision" or ("Artificial" and "Intelligence"))).mp. [mp=title, book title, abstract, original title, name of substance word, subject heading word, floating sub-heading word, keyword heading word, organism supplementary concept word, protocol supplementary concept word, rare disease supplementary concept word, unique identifier, synonyms, population supplementary concept word, anatomy supplementary concept word] | N = 1789 |
| Pubmed | ((("Vertebral Compression Fractures"[All Fields] OR "Spinal Fractures"[MeSH Terms] OR "Compression Fractures"[All Fields] OR "Vertebral"[All Fields] OR "Vertebral Fracture*"[All Fields]) OR (("Vertebral"[All Fields] AND "Compression"[All Fields]) AND ("Fracture*"[All Fields] OR "Trauma"[All Fields]))) AND (("Algorithms"[MeSH Terms] OR "Artificial Intelligence"[MeSH Terms] OR "AI"[All Fields] OR "Machine Learning"[MeSH Terms] OR "Neural Networks, Computer"[MeSH Terms] OR "Random Forest"[MeSH Terms] OR "artificial neural network"[All Fields] OR "ANN"[All Fields] OR "Support Vector Machine"[MeSH Terms] OR "support vector machine"[All Fields] OR "SVM"[All Fields] OR "random forest"[All Fields] OR "gradient boosting"[All Fields] OR "Decision Trees"[MeSH Terms] OR "decision tree"[All Fields] OR "convolutional neural network"[All Fields] OR "CNN"[All Fields] OR "Algorithm*"[All Fields] OR "Computer Vision"[All Fields]) OR ("Artificial"[All Fields] AND "Intelligence"))) | N = 3961 |
| Web of Science | (("Vertebral Compression Fractures" or "Spinal Fractures" or "Compression Fractures" or "Vertebral" or "Vertebral Fracture*" or ("Vertebral" and "Compression" and "Fracture*OR Trauma")) and ("Algorithms" or "Artificial Intelligence" or "AI" or "Machine Learning" or "Neural Networks, Computer" or "Random Forest" or "artificial neural network" or "ANN" or "Support Vector Machine" or "support vector machine" or "SVM" or "random forest" or "gradient boosting" or "Decision Trees" or "decision tree" or "convolutional neural network" or "CNN" or "Algorithm*" or "Computer Vision" or ("Artificial" and "Intelligence"))) | N = 4470 |
| Scopus | TITLE-ABS-KEY(("Vertebral Compression Fractures" OR "Spinal Fractures" OR "Compression Fractures" OR "Vertebral" OR "Vertebral Fracture*") AND ("Artificial Intelligence" OR "AI" OR "Machine Learning" OR "Neural Networks" OR "Random Forest" OR "Support Vector Machine" OR "SVM" OR "Gradient Boosting" OR "Decision Trees" OR "Convolutional Neural Network" OR "Computer Vision")) | N = 1076 |

**Supplementary Table 2:** Inclusion and exclusion criteria.

| Inclusion Criteria | Exclusion criteria |  |
| --- | --- | --- |
| - Published in peer reviewed journal - Published in the English language - Adult human patients with vertebral fractures - Studies that use AI/ML models to diagnose or predict the risk of fractures - Studies that focus on vertebral fractures - For the meta-analysis: Studies reporting the performance of their AI model (AUROC) | - Commentaries, case reports, letters to editors, narrative reviews, books - Studies which did not evaluate their AI performance |  |
|  |  |  |
|  |  |  |
|  |  |  |
|  |  |  |

**Supplementary Table 3:** complete PROBAST assessment, including the specific risk of bias scores for each study.

| **Author, AI model (if applicable), year** | **1.1** | **1.2** | **2.1** | **2.2** | **2.3** | **3.1** | **3.2** | **3.3** | **3.4** | **3.5** | **3.6** | **4.1** | **4.2** | **4.3** | **4.4** | **4.5** | **4.6** | **4.7** | **4.8** | **4.9** | **Risk of Bias** |
| --- | --- | --- | --- | --- | --- | --- | --- | --- | --- | --- | --- | --- | --- | --- | --- | --- | --- | --- | --- | --- | --- |
| Bodden J et al, (CNN), 2023 | High RoB | High RoB | High RoB | Unclear | High RoB | Low RoB | Low RoB | Low RoB | High RoB | Low RoB | High RoB | Low RoB | High RoB | Low RoB | Low RoB | Low RoB | Low RoB | Low RoB | Unclear | Unclear | High RoB |
| Sung Tan Cho et al, (Multivariate logistic regression), 2023 | Unclear | Low RoB | Low RoB | Unclear | Low RoB | Low RoB | Low RoB | Low RoB | Low RoB | High RoB | Unclear | Low RoB | Low RoB | Unclear | Low RoB | High RoB | Unclear | High RoB | Low RoB | High RoB | High RoB |
| Sung Tan Cho et al, (Decision Tree), 2023 | Low RoB | Unclear | Low RoB | Low RoB | Low RoB | Low RoB | Unclear | Unclear | Unclear | Unclear | Unclear | Unclear | Unclear | Unclear | Low RoB | Unclear | Unclear | Unclear | Unclear | High RoB | Unclear |
| Sung Tan Cho et al, (Random Forest), 2023 | Unclear | Unclear | Unclear | Low RoB | Low RoB | Low RoB | Unclear | Low RoB | Unclear | Low RoB | Low RoB | Unclear | Low RoB | Unclear | Unclear | Unclear | Unclear | Unclear | Unclear | High RoB | Unclear |
| Chen et al, (DCNN), 2023 | Unclear | Low RoB | Unclear | Unclear | Unclear | Low RoB | Unclear | Low RoB | Low RoB | Unclear | Unclear | Low RoB | Unclear | Unclear | Unclear | Low RoB | Unclear | Unclear | High RoB | Low RoB | Unclear |
| Chen et al, (Artificial detection combined with DCNN), 2023 | High RoB | High RoB | Low RoB | Low RoB | High RoB | High RoB | High RoB | High RoB | Unclear | High RoB | Low RoB | Low RoB | Low RoB | High RoB | Low RoB | Low RoB | Low RoB | Low RoB | Unclear | High RoB | High RoB |
| Taeyong Park, Min A Yoon et al, (CNN), 2022 | Low RoB | Unclear | Low RoB | Low RoB | Unclear | Unclear | Unclear | Unclear | Low RoB | Unclear | Low RoB | Unclear | Low RoB | Unclear | Unclear | Low RoB | Low RoB | Low RoB | Low RoB | Low RoB | Low RoB |
| Ma et al, (Logistic regression), 2023 | Unclear | Low RoB | Low RoB | Unclear | Unclear | Unclear | Unclear | Unclear | Unclear | Low RoB | Unclear | Low RoB | Unclear | Unclear | Unclear | Low RoB | Unclear | Unclear | Low RoB | Low RoB | Low RoB |
| Ma et al, (Random Forest), 2023 | Low RoB | Unclear | High RoB | High RoB | Low RoB | Low RoB | Low RoB | Unclear | High RoB | Low RoB | High RoB | Low RoB | High RoB | Unclear | Unclear | Unclear | Low RoB | Low RoB | High RoB | Low RoB | High RoB |
| Ma et al, (Gradient boosting machine ), 2023 | High RoB | Low RoB | Low RoB | High RoB | Low RoB | High RoB | Low RoB | High RoB | High RoB | High RoB | High RoB | Unclear | Unclear | High RoB | High RoB | Unclear | Low RoB | High RoB | Low RoB | Unclear | High RoB |
| Ma et al, (Decision tree), 2023 | Low RoB | Unclear | Low RoB | High RoB | High RoB | Low RoB | High RoB | High RoB | High RoB | High RoB | Low RoB | High RoB | Unclear | High RoB | High RoB | Unclear | Unclear | Unclear | Low RoB | High RoB | High RoB |
| Ma et al, (Support vector machine), 2023 | High RoB | Low RoB | Unclear | High RoB | Low RoB | Low RoB | Low RoB | High RoB | High RoB | Low RoB | High RoB | Low RoB | Unclear | Unclear | High RoB | High RoB | Low RoB | Low RoB | Low RoB | Unclear | High RoB |
| Ma et al, (Neural network), 2023 | Unclear | Unclear | Unclear | Unclear | Low RoB | Low RoB | Low RoB | High RoB | Low RoB | High RoB | Unclear | Low RoB | Low RoB | Low RoB | Unclear | Low RoB | Low RoB | Low RoB | High RoB | Unclear | High RoB |
| Ma et al, (Regularized discriminant analysis), 2023 | Low RoB | Unclear | Low RoB | Low RoB | Low RoB | Unclear | Low RoB | Low RoB | Unclear | Unclear | Low RoB | Unclear | Unclear | Low RoB | Low RoB | High RoB | Low RoB | Low RoB | Low RoB | Low RoB | Unclear |
| Chengcheng Gui et al, (Random forest classification models - SINS components), 2022 | Unclear | Unclear | Low RoB | Unclear | Low RoB | Unclear | Unclear | Low RoB | Unclear | Unclear | Unclear | Low RoB | Unclear | Unclear | Low RoB | Low RoB | Low RoB | High RoB | Low RoB | Low RoB | Unclear |
| Chengcheng Gui et al, (Random forest classification models - Selected clinical features alone), 2022 | Low RoB | Low RoB | Low RoB | Low RoB | Unclear | Low RoB | Low RoB | Low RoB | Unclear | Unclear | Low RoB | Unclear | Low RoB | Unclear | Unclear | Low RoB | Unclear | Low RoB | Unclear | Unclear | Low RoB |
| Chengcheng Gui et al, (Random forest classification models - Selected CT & T1W MR features), 2022 | Low RoB | Unclear | Unclear | Unclear | Unclear | Unclear | Unclear | Unclear | Low RoB | Unclear | Unclear | Low RoB | Low RoB | Low RoB | Low RoB | Low RoB | Unclear | Low RoB | Unclear | High RoB | Unclear |
| Chengcheng Gui et al, (Random forest classification models - Selected CT, T1W MR, & clinical features), 2022 | Unclear | Unclear | Unclear | Low RoB | Unclear | Unclear | Unclear | Low RoB | Unclear | Unclear | Low RoB | Low RoB | Unclear | Low RoB | Unclear | Unclear | Unclear | Unclear | Low RoB | High RoB | Unclear |
| Yunji Seol et al, (Gradient boosting), 2023 | Low RoB | Low RoB | Unclear | Low RoB | Unclear | Unclear | Unclear | Low RoB | Unclear | Unclear | Low RoB | Unclear | Low RoB | Low RoB | Low RoB | Unclear | Unclear | Unclear | Unclear | High RoB | Unclear |
| Xiao Hu et al, (CNN using the Xception module), 2022 | Low RoB | Low RoB | Unclear | Unclear | Unclear | Low RoB | Low RoB | Unclear | Unclear | Unclear | Unclear | Unclear | Unclear | Unclear | Unclear | Low RoB | Unclear | Low RoB | Low RoB | Unclear | Low RoB |
| Sung Hye Kong et al, (CNN with key-point detection), 2020 | Low RoB | High RoB | Low RoB | Unclear | Low RoB | High RoB | High RoB | Low RoB | High RoB | High RoB | Low RoB | Unclear | Low RoB | Low RoB | Unclear | Low RoB | Unclear | Unclear | Low RoB | Low RoB | High RoB |
| Sung Hye Kong et al, (CNN with Segmented Images), 2020 | Low RoB | Low RoB | High RoB | Low RoB | High RoB | Low RoB | High RoB | Low RoB | High RoB | Low RoB | High RoB | High RoB | High RoB | Low RoB | Low RoB | Low RoB | High RoB | Low RoB | Low RoB | Unclear | High RoB |
| Sung Hye Kong et al, (Naïve-Bayes Model), 2020 | High RoB | Low RoB | Unclear | Unclear | Unclear | Unclear | Low RoB | Low RoB | Low RoB | Low RoB | High RoB | High RoB | Low RoB | Low RoB | High RoB | Unclear | Unclear | Low RoB | Unclear | Low RoB | High RoB |
| Li Shen et al, (AI_OVF_SH - internal), 2023 | Unclear | Unclear | Unclear | Low RoB | Low RoB | Low RoB | Low RoB | Low RoB | Low RoB | Unclear | High RoB | Low RoB | Unclear | Low RoB | High RoB | Low RoB | High RoB | Low RoB | High RoB | Unclear | High RoB |
| Li Shen et al, (AI_OVF_SH - external), 2023 | High RoB | High RoB | Unclear | Unclear | High RoB | Unclear | Low RoB | Unclear | Low RoB | Unclear | High RoB | High RoB | Low RoB | Unclear | Unclear | High RoB | Low RoB | Low RoB | High RoB | Unclear | High RoB |
| Namki Hong et al, (VERTE-X pVF score - internal), 2023 | Low RoB | Unclear | High RoB | Low RoB | Low RoB | Unclear | High RoB | Low RoB | High RoB | Low RoB | Low RoB | Low RoB | Low RoB | High RoB | High RoB | Low RoB | High RoB | Unclear | Low RoB | Unclear | High RoB |
| Namki Hong et al, (VERTE-X pVF score - external), 2023 | Low RoB | High RoB | High RoB | Low RoB | High RoB | Low RoB | High RoB | High RoB | High RoB | High RoB | Unclear | Low RoB | High RoB | High RoB | Low RoB | Low RoB | Unclear | Low RoB | Unclear | Low RoB | High RoB |
| Namki Hong et al, (VERTE-X osteo score - internal), 2023 | High RoB | High RoB | Low RoB | Low RoB | Low RoB | High RoB | Low RoB | Unclear | High RoB | Low RoB | Low RoB | Low RoB | Low RoB | Unclear | High RoB | Low RoB | Unclear | Low RoB | Low RoB | High RoB | High RoB |
| Namki Hong et al, (VERTE-X osteo score - external), 2023 | Unclear | Low RoB | Unclear | Low RoB | Low RoB | High RoB | High RoB | Low RoB | Low RoB | High RoB | Low RoB | Low RoB | High RoB | High RoB | Unclear | Low RoB | High RoB | High RoB | Unclear | High RoB | High RoB |
| Del Lama et al, (MLP), 2022 | High RoB | Low RoB | Low RoB | High RoB | High RoB | High RoB | High RoB | High RoB | Low RoB | Low RoB | Unclear | Unclear | Low RoB | Low RoB | High RoB | Unclear | High RoB | High RoB | Unclear | Low RoB | High RoB |
| Del Lama et al, (CNN manually optimised), 2022 | Low RoB | Low RoB | Unclear | Low RoB | Low RoB | Unclear | Low RoB | Low RoB | Unclear | Low RoB | Low RoB | Unclear | Unclear | Unclear | Low RoB | Unclear | Low RoB | Unclear | High RoB | Low RoB | Unclear |
| Del Lama et al, (CNN manually optimised with data augmentation), 2022 | High RoB | High RoB | Low RoB | Unclear | High RoB | Low RoB | Low RoB | Low RoB | Low RoB | Low RoB | Low RoB | Low RoB | Low RoB | Low RoB | Low RoB | Low RoB | Unclear | Unclear | Low RoB | High RoB | High RoB |
| Del Lama et al, (Pre-trained CNN), 2022 | Low RoB | High RoB | High RoB | Unclear | Unclear | Low RoB | Low RoB | Unclear | Low RoB | High RoB | Unclear | Low RoB | High RoB | High RoB | High RoB | High RoB | High RoB | High RoB | High RoB | High RoB | High RoB |
| Del Lama et al, (Hybrid model using pre-trained CNN), 2022 | Low RoB | Unclear | Unclear | Unclear | Unclear | Unclear | Unclear | Low RoB | Unclear | Unclear | Low RoB | Low RoB | Low RoB | Low RoB | Unclear | Unclear | Unclear | Unclear | High RoB | Low RoB | Unclear |
| Del Lama et al, (Hybrid model optimised by the GA), 2022 | Unclear | Unclear | Unclear | Low RoB | Unclear | Unclear | Unclear | Unclear | Low RoB | Unclear | Unclear | Unclear | Low RoB | Unclear | Unclear | Unclear | Unclear | Low RoB | Unclear | Low RoB | Low RoB |
| J.E. Small et al, (CNN), 2021 | High RoB | High RoB | Low RoB | Unclear | Low RoB | Low RoB | Low RoB | Unclear | Unclear | Unclear | Low RoB | Low RoB | Low RoB | Low RoB | Low RoB | Low RoB | Unclear | High RoB | Low RoB | Unclear | High RoB |
| Akito Yabu et al, (VGG16, VGG19, DenseNet201, ResNet50), 2021 | Low RoB | Unclear | Low RoB | Unclear | Low RoB | Low RoB | Unclear | Unclear | Unclear | Unclear | Unclear | Low RoB | Unclear | Low RoB | Unclear | Low RoB | Low RoB | Unclear | Low RoB | High RoB | Unclear |
| Jae Won Seo et al, (CNN), 2021 | Low RoB | Low RoB | Low RoB | High RoB | Unclear | High RoB | Low RoB | Low RoB | Low RoB | High RoB | High RoB | High RoB | Low RoB | Low RoB | Low RoB | High RoB | High RoB | Low RoB | Low RoB | Unclear | High RoB |
| Kazuma Murata et al, (DCNN), 2020 | Low RoB | Unclear | Low RoB | Low RoB | Low RoB | Low RoB | Low RoB | Low RoB | Low RoB | Low RoB | Low RoB | Unclear | Unclear | High RoB | Low RoB | Low RoB | Low RoB | Unclear | Low RoB | Low RoB | Unclear |
| Weijuan Chen et al, (Deep Learning), 2021 | Unclear | Unclear | Unclear | Low RoB | Low RoB | Low RoB | Low RoB | Low RoB | Low RoB | Unclear | Unclear | Low RoB | Low RoB | Unclear | Unclear | Low RoB | Low RoB | High RoB | Unclear | Unclear | Unclear |
| Yuan Li et al, (ResNet50), 2021 | Low RoB | Low RoB | Low RoB | Unclear | Low RoB | Low RoB | Unclear | Low RoB | Low RoB | Low RoB | Unclear | Unclear | Low RoB | Unclear | Low RoB | Unclear | Low RoB | Unclear | Low RoB | High RoB | Unclear |
| Dong Hyun Kim et al, (Deep Learning). 2021 | Unclear | Low RoB | Unclear | Low RoB | Unclear | Unclear | Unclear | Low RoB | Unclear | Unclear | Unclear | Low RoB | Unclear | Low RoB | Low RoB | Low RoB | Unclear | Unclear | Unclear | Low RoB | Low RoB |
| Alena-Kathrin Golla et al, 2023 | High RoB | Low RoB | Low RoB | High RoB | Low RoB | High RoB | Unclear | Low RoB | Unclear | Low RoB | Low RoB | High RoB | Low RoB | Low RoB | Low RoB | Low RoB | Unclear | Unclear | High RoB | Low RoB | High RoB |
| Hsuan-Yu Chen et al, (DCNN), 2021 | Unclear | Low RoB | Low RoB | Unclear | Low RoB | Unclear | Low RoB | Unclear | Unclear | Unclear | Unclear | Unclear | Unclear | Unclear | Unclear | Unclear | Unclear | Unclear | High RoB | Unclear | Unclear |
| E. Biamonte et al, (radiomics) 2022 | Unclear | Low RoB | Low RoB | Low RoB | High RoB | Low RoB | Low RoB | High RoB | Low RoB | Unclear | Unclear | Unclear | Low RoB | Low RoB | Unclear | Low RoB | Low RoB | Low RoB | High RoB | Unclear | High RoB |
| Naofumi Tomita, (CNN), 2018 | Low RoB | Unclear | Low RoB | Unclear | Low RoB | Unclear | Unclear | Unclear | Low RoB | Low RoB | Low RoB | Low RoB | Unclear | Unclear | Unclear | High RoB | Unclear | Low RoB | Low RoB | Unclear | Unclear |
| Fan Xu et al, (GZFPH dataset), 2023 | Low RoB | Unclear | Low RoB | Low RoB | Unclear | Low RoB | Low RoB | Low RoB | Unclear | Low RoB | Unclear | Unclear | Low RoB | Unclear | Unclear | Unclear | Unclear | Unclear | Low RoB | Unclear | Low RoB |
| Fan Xu et al, (WHTH dataset), 2023 | Low RoB | Low RoB | Unclear | Unclear | Low RoB | Low RoB | Low RoB | Unclear | Unclear | Low RoB | Low RoB | Low RoB | Low RoB | Unclear | Unclear | Unclear | Unclear | Unclear | High RoB | Low RoB | Unclear |
| Fan Xu et al, (HB672H dataset), 2023 | Low RoB | Unclear | Low RoB | Unclear | Unclear | Low RoB | Low RoB | Unclear | Low RoB | Low RoB | Unclear | Unclear | Unclear | Unclear | Low RoB | Unclear | Low RoB | Unclear | Unclear | Unclear | Low RoB |
| Yurim Lee et al, (DCNN), 2023 | Unclear | Unclear | Unclear | Unclear | Low RoB | Unclear | Unclear | Low RoB | Unclear | Low RoB | Low RoB | Unclear | Unclear | Unclear | Low RoB | Low RoB | High RoB | Low RoB | Unclear | Low RoB | Unclear |
| A.F. Voter, (Aidoc). 2021 | Unclear | Low RoB | Unclear | Low RoB | Low RoB | Unclear | Low RoB | Unclear | High RoB | High RoB | Low RoB | Low RoB | Low RoB | Low RoB | Low RoB | Unclear | Low RoB | Low RoB | Low RoB | Low RoB | High RoB |
| Jinhui Cai, (Combined-LR), 2023 | High RoB | High RoB | Unclear | Low RoB | Low RoB | High RoB | Low RoB | Unclear | Low RoB | High RoB | High RoB | High RoB | Unclear | Low RoB | Low RoB | Unclear | Low RoB | Low RoB | Low RoB | High RoB | High RoB |
| Jinhui Cai, (Combined-RF), 2023 | Unclear | Unclear | Low RoB | Unclear | Unclear | Unclear | Unclear | Unclear | Low RoB | Low RoB | Unclear | Low RoB | Low RoB | Low RoB | Low RoB | High RoB | Low RoB | Low RoB | Low RoB | Low RoB | Unclear |
| Jinhui Cai, (Combined-SVM 0), 2023 | High RoB | Unclear | High RoB | High RoB | Unclear | Unclear | High RoB | Low RoB | Low RoB | Low RoB | Low RoB | Low RoB | High RoB | High RoB | High RoB | Low RoB | High RoB | Low RoB | Low RoB | Low RoB | High RoB |
| Jinhui Cai, (Combined-XGBoost), 2023 | Unclear | Low RoB | Unclear | Low RoB | Unclear | Unclear | Low RoB | Low RoB | Low RoB | Low RoB | Unclear | Low RoB | Low RoB | Unclear | Unclear | Low RoB | Unclear | Low RoB | Low RoB | Low RoB | Low RoB |
| Barret A. Monchka et al, (Single-energy), 2021 | Low RoB | Unclear | Unclear | Unclear | Low RoB | Unclear | Unclear | Unclear | Unclear | Low RoB | Unclear | Unclear | Low RoB | Low RoB | Unclear | Unclear | High RoB | Low RoB | Low RoB | Low RoB | Unclear |
| Barret A. Monchka et al, (Composite), 2021 | Unclear | Unclear | Unclear | Low RoB | Low RoB | Unclear | Unclear | Unclear | Unclear | Unclear | Unclear | Low RoB | Low RoB | Low RoB | Unclear | Low RoB | Low RoB | High RoB | Low RoB | Low RoB | Unclear |
| Barret A. Monchka et al, (Dual-energy), 2021 | Low RoB | Unclear | Low RoB | Unclear | Low RoB | Unclear | Low RoB | Low RoB | Low RoB | Unclear | Low RoB | Low RoB | Low RoB | Low RoB | Low RoB | Unclear | Low RoB | High RoB | Low RoB | Low RoB | Unclear |
| Barret A. Monchka et al, (Single-energy), 2021** | High RoB | Low RoB | Low RoB | Unclear | Low RoB | Low RoB | Low RoB | Low RoB | Low RoB | Unclear | Low RoB | Low RoB | Unclear | Unclear | Low RoB | Low RoB | Unclear | Unclear | Low RoB | Unclear | Unclear |
| Barret A. Monchka et al, (Composite), 2021** | Unclear | Low RoB | Low RoB | Low RoB | Low RoB | Low RoB | High RoB | High RoB | Unclear | Unclear | Unclear | Low RoB | High RoB | High RoB | Unclear | High RoB | High RoB | High RoB | High RoB | High RoB | High RoB |
| Barret A. Monchka et al, (Dual-energy), 2021** | High RoB | Unclear | Unclear | Unclear | Low RoB | Unclear | Low RoB | Unclear | High RoB | High RoB | Unclear | Unclear | Unclear | Low RoB | Low RoB | High RoB | High RoB | High RoB | High RoB | Unclear | High RoB |
| Yi-Chu Li MS et al, 2021 | Low RoB | Low RoB | Low RoB | Low RoB | Low RoB | High RoB | Low RoB | High RoB | Low RoB | Low RoB | High RoB | High RoB | Low RoB | Low RoB | High RoB | High RoB | Low RoB | Low RoB | High RoB | High RoB | High RoB |
| Yoda et al, (STIR CNN), 2022 | Low RoB | Low RoB | Unclear | Low RoB | Low RoB | High RoB | Low RoB | Unclear | Low RoB | Low RoB | Low RoB | Low RoB | Low RoB | Low RoB | Unclear | Low RoB | High RoB | Low RoB | Low RoB | High RoB | High RoB |
| Yoda et al, (T1WI CNN), 2022 | Unclear | Unclear | High RoB | Low RoB | High RoB | Low RoB | Unclear | Low RoB | Low RoB | High RoB | Low RoB | Unclear | Unclear | Low RoB | Low RoB | High RoB | Unclear | Low RoB | Low RoB | Unclear | High RoB |
| Barret A. Monchka, et al, (CNN OsteoLaus Study data set Hologic VFA type), 2022 | Unclear | Low RoB | Low RoB | Low RoB | Low RoB | Low RoB | Unclear | Low RoB | Unclear | Unclear | Low RoB | Unclear | Unclear | Low RoB | Low RoB | Low RoB | Unclear | Low RoB | Low RoB | Unclear | Low RoB |
| Barret A. Monchka, et al, (CNN Manitoba data set GE dual-energy VFA type), 2022 | Low RoB | Low RoB | High RoB | Unclear | Low RoB | High RoB | Low RoB | High RoB | High RoB | High RoB | Low RoB | High RoB | Low RoB | Low RoB | Low RoB | Unclear | Low RoB | Low RoB | Unclear | High RoB | High RoB |
| Barret A. Monchka, et al, (CNN Manitoba data set GE single-energy VFA type), 2022 | Low RoB | Unclear | Unclear | Unclear | Unclear | Unclear | Unclear | Unclear | Unclear | Unclear | Unclear | Low RoB | Unclear | Unclear | Low RoB | Low RoB | Unclear | Unclear | Unclear | High RoB | Unclear |
| Barret A. Monchka, et al, (CNN OsteoLaus Study data set Hologic VFA type), 2022** | Low RoB | Low RoB | High RoB | Low RoB | High RoB | High RoB | High RoB | High RoB | Low RoB | Low RoB | Low RoB | Low RoB | High RoB | High RoB | Low RoB | Unclear | Low RoB | Low RoB | Low RoB | High RoB | High RoB |
| Barret A. Monchka, et al, (CNN Manitoba data set GE dual-energy VFA type), 2022** | Unclear | Unclear | Unclear | Unclear | Unclear | Low RoB | Unclear | Unclear | Low RoB | Unclear | Low RoB | Unclear | Unclear | Unclear | Low RoB | Unclear | Low RoB | Unclear | Low RoB | High RoB | Unclear |
| Barret A. Monchka, et al, (Mean ensemble CNN Manitoba data set GE single-energy VFA type), 2022** | Unclear | Unclear | Unclear | Low RoB | Unclear | Low RoB | Unclear | Low RoB | Low RoB | Unclear | Unclear | Unclear | Low RoB | Low RoB | Unclear | Low RoB | Low RoB | High RoB | Low RoB | Low RoB | Unclear |
| Christoph Germann et al, (DCNN), 2023 | Low RoB | Low RoB | Low RoB | Low RoB | Low RoB | Low RoB | Low RoB | Unclear | Unclear | Low RoB | Unclear | Low RoB | Unclear | Unclear | Unclear | Low RoB | Unclear | High RoB | Low RoB | Low RoB | Unclear |
| Zhang J et al, (multistage AO system), 2023 | Low RoB | High RoB | Low RoB | Unclear | High RoB | Low RoB | Low RoB | Unclear | Low RoB | Low RoB | Low RoB | Low RoB | Unclear | Low RoB | Low RoB | Unclear | Unclear | High RoB | High RoB | Low RoB | High RoB |
| Wu-Gen Li et al, (SVM), 2023 | Low RoB | Low RoB | Unclear | Unclear | Low RoB | Unclear | Unclear | Unclear | Unclear | Unclear | Low RoB | Low RoB | Low RoB | Low RoB | Low RoB | Low RoB | High RoB | Low RoB | Unclear | Low RoB | Unclear |
| Wu-Gen Li et al, (LR), 2023 | Low RoB | Unclear | Low RoB | Low RoB | Unclear | Unclear | Unclear | Unclear | Low RoB | Unclear | Low RoB | Low RoB | Low RoB | Unclear | Unclear | Low RoB | Unclear | High RoB | Unclear | Low RoB | Unclear |
| Wu-Gen Li et al, (Sagittal), 2023 | Unclear | Low RoB | Unclear | Unclear | Unclear | Unclear | Unclear | Unclear | Low RoB | Low RoB | Unclear | Unclear | Unclear | Unclear | Unclear | Unclear | Low RoB | Unclear | Unclear | High RoB | Unclear |
| Wu-Gen Li et al, (SVM), 2023** | Unclear | Low RoB | Unclear | High RoB | Unclear | Low RoB | Unclear | High RoB | Low RoB | Low RoB | Low RoB | Low RoB | Low RoB | High RoB | Unclear | Unclear | High RoB | High RoB | Low RoB | Low RoB | High RoB |
| Wu-Gen Li et al, (LR), 2023** | High RoB | High RoB | Unclear | Unclear | Unclear | Low RoB | Low RoB | Low RoB | Low RoB | Low RoB | High RoB | Unclear | Low RoB | High RoB | High RoB | Unclear | Low RoB | High RoB | Unclear | Low RoB | High RoB |
| Wu-Gen Li et al, (Sagittal), 2023** | Unclear | High RoB | High RoB | High RoB | Unclear | Low RoB | Low RoB | Low RoB | Low RoB | Unclear | High RoB | Low RoB | Unclear | Low RoB | Low RoB | Low RoB | Low RoB | High RoB | High RoB | High RoB | High RoB |
| Natália S. Chiari-Correia et al, (ANN), 2023 | Low RoB | Unclear | Low RoB | Low RoB | Unclear | Unclear | Unclear | Low RoB | Unclear | Low RoB | Low RoB | Low RoB | Low RoB | Unclear | Unclear | Unclear | Unclear | Low RoB | Low RoB | Unclear | Low RoB |
| Cristina Eller-Vainicher et al, (CNN SDI>1), 2011 | Low RoB | Unclear | Low RoB | Low RoB | Low RoB | Unclear | Unclear | Unclear | Low RoB | Low RoB | Unclear | Low RoB | Unclear | Low RoB | Low RoB | Unclear | Unclear | Low RoB | Unclear | Low RoB | Low RoB |
| Cristina Eller-Vainicher et al, (CNN SDI<1), 2011 | Low RoB | Unclear | Low RoB | Low RoB | Unclear | Unclear | Unclear | Low RoB | Unclear | Unclear | Low RoB | Low RoB | Unclear | Unclear | Low RoB | Low RoB | Unclear | Unclear | Unclear | High RoB | Unclear |
| Beibei Liu et al, (TSCCN - T1WIT1), 2023 | Unclear | Low RoB | Unclear | Low RoB | Unclear | Low RoB | Unclear | Low RoB | Low RoB | Low RoB | Unclear | Low RoB | Low RoB | Unclear | Low RoB | Unclear | Unclear | Low RoB | Unclear | High RoB | Unclear |
| Beibei Liu et al, (TSCCN -T2WI-FS T2-weighted fat suppression), 2023 | Unclear | Low RoB | Unclear | Unclear | Low RoB | Low RoB | Low RoB | Unclear | Low RoB | Low RoB | Low RoB | Unclear | Unclear | High RoB | Low RoB | Low RoB | Unclear | Low RoB | Low RoB | Low RoB | Unclear |
| Beibei Liu et al, (TSCCN - T1WI/T2WI-FS), 2023 | Unclear | High RoB | Unclear | High RoB | Low RoB | High RoB | Low RoB | High RoB | Low RoB | High RoB | Low RoB | High RoB | High RoB | Unclear | Unclear | Low RoB | High RoB | High RoB | Unclear | High RoB | High RoB |
| Urs J. Muehlematter et al, (SVM), 2019 | Unclear | Unclear | Low RoB | Unclear | Unclear | Low RoB | Unclear | Low RoB | Unclear | Unclear | Unclear | Unclear | Low RoB | Low RoB | Unclear | Low RoB | Low RoB | High RoB | Low RoB | Unclear | Unclear |
| Joeri Nicolaes et al, (SQ23 (normal/mild versus moderate/severe) - Subject level), 2023 | Low RoB | Unclear | Unclear | Low RoB | Low RoB | Unclear | Low RoB | Unclear | Unclear | Low RoB | Low RoB | Low RoB | Unclear | Low RoB | Unclear | Low RoB | Low RoB | High RoB | Low RoB | Unclear | Unclear |
| Joeri Nicolaes et al, (SQ23 (normal/mild versus moderate/severe) - Vertebral level) - Subject level), 2023 | Unclear | Unclear | Low RoB | Unclear | Unclear | Unclear | Unclear | Low RoB | Unclear | Low RoB | Unclear | Low RoB | Unclear | Unclear | Low RoB | Unclear | High RoB | Unclear | Low RoB | Unclear | Unclear |
| Joeri Nicolaes et al, (SQ123 (normal versus mild/moderate/severe) outcome - Subject level), 2023** | Unclear | Low RoB | Unclear | Low RoB | Low RoB | Unclear | Unclear | Unclear | Unclear | Unclear | Unclear | Unclear | Unclear | Low RoB | Unclear | Unclear | Low RoB | High RoB | Low RoB | Unclear | Unclear |
| Joeri Nicolaes et al, (SQ123 (normal versus mild/moderate/severe) outcome - Vertebral level, 2023** | Low RoB | Low RoB | Unclear | Low RoB | Unclear | Low RoB | Low RoB | Unclear | Unclear | Unclear | Low RoB | Unclear | High RoB | Unclear | Low RoB | Low RoB | Low RoB | Low RoB | Unclear | Low RoB | Unclear |
| Joeri Nicolaes et al, (SQ23 (normal/mild versus moderate/severe) - Subject level), 2024 | High RoB | Unclear | Unclear | Low RoB | High RoB | High RoB | Unclear | High RoB | Unclear | Low RoB | Low RoB | Unclear | Low RoB | Low RoB | Low RoB | Low RoB | High RoB | Unclear | Low RoB | Low RoB | High RoB |
| Joeri Nicolaes et al, (SQ23 (normal/mild versus moderate/severe) - Vertebral level) - Subject level), 2024 | High RoB | Low RoB | Low RoB | Low RoB | Unclear | Unclear | Low RoB | Low RoB | Unclear | High RoB | Unclear | Unclear | Low RoB | Unclear | Unclear | Low RoB | High RoB | Unclear | High RoB | Low RoB | High RoB |
| Joeri Nicolaes et al, (SQ123 (normal versus mild/moderate/severe) outcome - Subject level), 2024** | Unclear | Unclear | Unclear | Low RoB | Unclear | Low RoB | High RoB | Unclear | Unclear | Low RoB | Low RoB | Low RoB | Low RoB | Low RoB | Unclear | High RoB | Unclear | Low RoB | High RoB | Low RoB | High RoB |
| Joeri Nicolaes et al, (SQ123 (normal versus mild/moderate/severe) outcome - Vertebral level, 2024** | Unclear | Low RoB | Unclear | Unclear | Low RoB | Low RoB | Unclear | Unclear | Low RoB | Unclear | Unclear | Unclear | Unclear | Low RoB | Low RoB | Unclear | Low RoB | Unclear | Unclear | High RoB | Unclear |
| Shuo Duan et al, (DL), 2023 | Unclear | Low RoB | Unclear | Unclear | Low RoB | Unclear | Unclear | Unclear | Unclear | Unclear | Unclear | Unclear | Low RoB | Unclear | Low RoB | Unclear | Low RoB | Unclear | Unclear | Low RoB | Low RoB |
| Shuo Duan et al, (Rad), 2023 | Low RoB | Low RoB | Unclear | Unclear | Unclear | Unclear | Unclear | Low RoB | High RoB | Low RoB | Low RoB | Low RoB | Low RoB | Low RoB | Low RoB | Low RoB | Low RoB | Low RoB | Low RoB | Low RoB | High RoB |
| Shuo Duan et al, (DL_Rad), 2023 | Unclear | Unclear | Unclear | Low RoB | Unclear | Low RoB | Unclear | Low RoB | Low RoB | Low RoB | Low RoB | Unclear | Unclear | Low RoB | Unclear | Unclear | Low RoB | High RoB | Unclear | Low RoB | Unclear |
| Qifei Dong et al, (Task 2, Local-m2ABQ test), 2023 | High RoB | High RoB | Low RoB | High RoB | Low RoB | High RoB | Unclear | Low RoB | Unclear | High RoB | Low RoB | Low RoB | Unclear | Low RoB | Unclear | Unclear | Unclear | High RoB | High RoB | Low RoB | High RoB |
| Qifei Dong et al, (Task 2, MrOS-m2ABQ test), 2023 | Unclear | Low RoB | Low RoB | Low RoB | Low RoB | Low RoB | Low RoB | Low RoB | Unclear | Unclear | Unclear | Unclear | Unclear | Unclear | Unclear | Unclear | Low RoB | Unclear | Low RoB | Low RoB | Low RoB |
| Qifei Dong et al, (Task 3, Local-m2ABQ test), 2023 | High RoB | Low RoB | Unclear | Low RoB | High RoB | Unclear | High RoB | Low RoB | Unclear | High RoB | Unclear | High RoB | High RoB | High RoB | High RoB | Unclear | Unclear | Unclear | Low RoB | Low RoB | High RoB |
| Samah Al-Helo et al, (NN), 2012 | Unclear | Low RoB | Low RoB | High RoB | Low RoB | Low RoB | Low RoB | Low RoB | Low RoB | Low RoB | Low RoB | Unclear | High RoB | High RoB | Low RoB | Unclear | High RoB | Low RoB | High RoB | High RoB | High RoB |
| Samah Al-Helo et al, (NN), 2012 | Low RoB | Unclear | Low RoB | Low RoB | Unclear | Unclear | Unclear | Low RoB | Unclear | Unclear | Unclear | High RoB | Low RoB | Low RoB | Low RoB | Low RoB | Low RoB | Unclear | Low RoB | Low RoB | Unclear |
| Shrey K. Thawait et al, (Logistic Regression), 2013 | Unclear | Unclear | Unclear | Unclear | Unclear | Low RoB | Low RoB | Low RoB | Unclear | Unclear | Unclear | Unclear | Low RoB | Low RoB | Unclear | Unclear | Low RoB | Low RoB | High RoB | Unclear | Unclear |
| Shrey K. Thawait et al, (Classification Tree), 2013 | Low RoB | High RoB | High RoB | Unclear | Low RoB | High RoB | Unclear | Unclear | Low RoB | High RoB | High RoB | High RoB | Unclear | High RoB | High RoB | Unclear | High RoB | Unclear | High RoB | Low RoB | High RoB |
| Shrey K. Thawait et al, (Support Vector Machine), 2013 | Unclear | Low RoB | Low RoB | High RoB | Unclear | Low RoB | Unclear | Unclear | High RoB | Low RoB | High RoB | Unclear | Unclear | Unclear | Low RoB | High RoB | High RoB | Low RoB | High RoB | Low RoB | High RoB |
| Shrey K. Thawait et al, (Neural Network), 2013 | Low RoB | Low RoB | High RoB | Low RoB | Low RoB | Low RoB | Low RoB | Low RoB | High RoB | Unclear | Low RoB | Unclear | Low RoB | High RoB | Low RoB | Low RoB | Low RoB | Low RoB | Unclear | High RoB | High RoB |
| Euijoon Choi et al, (MULAN), 2023 | Low RoB | Unclear | Unclear | Low RoB | Low RoB | Low RoB | Low RoB | Low RoB | Unclear | Unclear | Low RoB | Low RoB | Unclear | Low RoB | Unclear | Low RoB | Unclear | Low RoB | High RoB | Low RoB | Unclear |
| Euijoon Choi et al, (Vertebral-level supervised), 2023 | Low RoB | Low RoB | Low RoB | Low RoB | High RoB | Low RoB | High RoB | Low RoB | High RoB | Unclear | High RoB | High RoB | Low RoB | Low RoB | Low RoB | High RoB | Low RoB | High RoB | Low RoB | High RoB | High RoB |
| Xun Wang et al, (SVM), 2023 | Unclear | Low RoB | Low RoB | Unclear | Unclear | Low RoB | Unclear | Unclear | Unclear | Low RoB | Unclear | Low RoB | Unclear | Unclear | Unclear | Low RoB | High RoB | Unclear | Low RoB | Low RoB | Unclear |
| Xun Wang et al, (Radiomics), 2023 | Unclear | Unclear | Unclear | Low RoB | Unclear | Unclear | Low RoB | Unclear | Low RoB | Low RoB | Low RoB | Unclear | Unclear | Unclear | Unclear | Unclear | High RoB | Low RoB | Unclear | Low RoB | Unclear |
| John H Page et al, 2023 | Low RoB | Unclear | Unclear | Unclear | Unclear | Low RoB | Low RoB | Unclear | Unclear | Low RoB | Low RoB | Unclear | Unclear | Low RoB | Unclear | Low RoB | Unclear | Unclear | Unclear | Low RoB | Low RoB |
| John H Page et al, (Zebra Medical Imaging), 2023 | High RoB | Unclear | Low RoB | Low RoB | Low RoB | Low RoB | High RoB | High RoB | Unclear | High RoB | Low RoB | Unclear | High RoB | Unclear | Low RoB | High RoB | Unclear | Unclear | Low RoB | Low RoB | High RoB |
| Jenna Silberstein et al, (Ofeye 1.0), 2023 | Low RoB | Low RoB | Unclear | Low RoB | Low RoB | Unclear | Low RoB | Unclear | Low RoB | Low RoB | Unclear | Low RoB | Unclear | Low RoB | Low RoB | Unclear | High RoB | Low RoB | Unclear | Unclear | Unclear |
| Yan-Ni Wang et al, (ResNet50), 2024 | Low RoB | High RoB | Unclear | Low RoB | High RoB | High RoB | Unclear | Low RoB | Low RoB | Low RoB | Unclear | Low RoB | Low RoB | Low RoB | High RoB | High RoB | Low RoB | High RoB | High RoB | Low RoB | High RoB |
| Xiao, B. et al, (Ofeye 1.0.), 2022 | Low RoB | High RoB | Low RoB | High RoB | High RoB | Low RoB | Unclear | Low RoB | High RoB | Unclear | High RoB | Unclear | High RoB | Unclear | High RoB | Low RoB | Low RoB | Unclear | Unclear | High RoB | High RoB |
| Li-Wei Cheng et all, (RF), 2024 | Unclear | Low RoB | High RoB | High RoB | High RoB | Low RoB | Low RoB | Low RoB | Unclear | Unclear | Unclear | Unclear | Low RoB | High RoB | Unclear | High RoB | High RoB | High RoB | Unclear | High RoB | High RoB |
| Li-Wei Cheng et all, (KNN), 2024 | Low RoB | Low RoB | Unclear | Low RoB | Low RoB | High RoB | Low RoB | Unclear | High RoB | Low RoB | Unclear | Unclear | Unclear | Unclear | Low RoB | High RoB | Low RoB | Unclear | High RoB | Unclear | High RoB |
| Li-Wei Cheng et all, (SVM), 2024 | Unclear | Unclear | Unclear | High RoB | Unclear | High RoB | High RoB | Low RoB | Unclear | Unclear | Low RoB | Low RoB | Unclear | High RoB | Low RoB | Low RoB | Low RoB | High RoB | Low RoB | Unclear | High RoB |
| Li-Wei Cheng et all, (MLP), 2024 | Low RoB | Low RoB | Unclear | Unclear | Low RoB | Unclear | Low RoB | Unclear | Low RoB | Low RoB | Low RoB | Low RoB | Low RoB | Unclear | Unclear | Low RoB | Unclear | Low RoB | Unclear | Unclear | Low RoB |
| Li-Wei Cheng et all, (XGBoost), 2024 | Unclear | Unclear | Unclear | Unclear | Unclear | Low RoB | Low RoB | Unclear | Low RoB | Unclear | Unclear | Low RoB | Unclear | Unclear | Unclear | Unclear | Low RoB | Low RoB | High RoB | Unclear | Unclear |
| Yohei Ono et al, (Resnet-50, DenseNet-161, and ResNeXt-50), 2023 | Unclear | Low RoB | Unclear | High RoB | Low RoB | Low RoB | High RoB | High RoB | Unclear | High RoB | High RoB | High RoB | High RoB | Unclear | High RoB | Low RoB | High RoB | Unclear | Unclear | Unclear | High RoB |
| Srinivasa Rao Gadu et al, (Hybrid UDA Net architecture), 2023 | Unclear | Low RoB | Low RoB | Unclear | Unclear | Low RoB | Unclear | Unclear | Low RoB | Low RoB | Unclear | Unclear | Unclear | Low RoB | Low RoB | Unclear | Unclear | High RoB | Unclear | Unclear | Unclear |
| Hao Zhang et al, (GNB), 2023 | Low RoB | Low RoB | Low RoB | Low RoB | High RoB | Low RoB | Unclear | Low RoB | High RoB | Low RoB | High RoB | Unclear | Unclear | Low RoB | High RoB | Low RoB | Low RoB | Low RoB | High RoB | Low RoB | High RoB |
| Hao Zhang et al, (KNN), 2023 | Unclear | Unclear | Unclear | Unclear | Low RoB | Unclear | Unclear | Low RoB | Unclear | Unclear | Low RoB | Low RoB | Unclear | Unclear | High RoB | Low RoB | Low RoB | Low RoB | Unclear | Unclear | Unclear |
| Hao Zhang et al, (LR), 2023 | Unclear | Low RoB | Low RoB | Unclear | Low RoB | High RoB | Low RoB | Low RoB | Low RoB | Unclear | Unclear | High RoB | High RoB | High RoB | Unclear | Low RoB | High RoB | High RoB | High RoB | High RoB | High RoB |
| Hao Zhang et al, (MLP), 2023 | Low RoB | High RoB | Unclear | Low RoB | Unclear | High RoB | High RoB | High RoB | Low RoB | Unclear | Unclear | Unclear | Low RoB | High RoB | High RoB | High RoB | Low RoB | Low RoB | Low RoB | High RoB | High RoB |
| Hao Zhang et al, (DT), 2023 | Unclear | Unclear | Unclear | Low RoB | Low RoB | Unclear | Unclear | Low RoB | Low RoB | Low RoB | Unclear | Low RoB | Low RoB | Unclear | Unclear | Unclear | High RoB | Low RoB | Unclear | Low RoB | Unclear |
| Hao Zhang et al, (LD), 2023 | Low RoB | High RoB | High RoB | High RoB | High RoB | High RoB | Unclear | Unclear | Low RoB | Low RoB | Low RoB | Low RoB | Unclear | Low RoB | High RoB | Low RoB | Low RoB | Low RoB | Low RoB | Low RoB | High RoB |
| Hao Zhang et al, (GB), 2023 | Unclear | Unclear | Low RoB | Low RoB | Unclear | Low RoB | Low RoB | Unclear | Unclear | Unclear | Unclear | Low RoB | Unclear | Low RoB | Low RoB | Low RoB | Low RoB | High RoB | Low RoB | Low RoB | Unclear |
| Seung Min Ryu et al, (ResNet101), 2023 | Low RoB | Unclear | Low RoB | Low RoB | Low RoB | Unclear | Low RoB | Low RoB | Unclear | Unclear | Unclear | Unclear | Low RoB | Low RoB | Low RoB | Unclear | Unclear | Unclear | Unclear | Low RoB | Low RoB |
| Eren B. Yilmaz et al, (CNN) , 2023 | Unclear | Unclear | Low RoB | Unclear | Unclear | Unclear | Unclear | Unclear | Unclear | Unclear | Unclear | Unclear | High RoB | Low RoB | Low RoB | Low RoB | Low RoB | Unclear | Low RoB | Low RoB | Unclear |
| Sankaran Iyer et al, (3-layered CNN), 2023 | Unclear | Low RoB | Low RoB | Unclear | Unclear | Unclear | Low RoB | Unclear | Low RoB | Unclear | Low RoB | Unclear | Unclear | Low RoB | Low RoB | Low RoB | Unclear | Unclear | Low RoB | Low RoB | Low RoB |
| Sankaran Iyer et al, (6-layered CNN), 2023 | Low RoB | Low RoB | Low RoB | Low RoB | High RoB | High RoB | High RoB | Low RoB | High RoB | Low RoB | High RoB | Low RoB | Low RoB | High RoB | Unclear | Unclear | Low RoB | Low RoB | Low RoB | Unclear | High RoB |
| Sankaran Iyer et al, (VGG16), 2023 | Low RoB | Unclear | High RoB | High RoB | High RoB | Low RoB | Unclear | High RoB | Unclear | Low RoB | Low RoB | Unclear | Low RoB | Unclear | Low RoB | Unclear | High RoB | High RoB | Low RoB | High RoB | High RoB |
| Sankaran Iyer et al, (ResNet50), 2023 | Unclear | Low RoB | Low RoB | Unclear | Low RoB | Low RoB | Low RoB | Unclear | High RoB | Low RoB | Low RoB | Unclear | Low RoB | Unclear | Low RoB | Low RoB | Low RoB | Low RoB | Low RoB | High RoB | High RoB |
| M. Bhargavi Sri Bhavya et al, (CNN), 2022 | Unclear | Low RoB | Low RoB | Unclear | Unclear | Unclear | Low RoB | Unclear | Low RoB | Unclear | Unclear | Low RoB | Low RoB | Low RoB | Unclear | Unclear | Low RoB | Unclear | Low RoB | Low RoB | Low RoB |
| Shixiang Feng et al, (TSCCN), 2021 | Unclear | Unclear | High RoB | High RoB | Unclear | Low RoB | High RoB | Low RoB | High RoB | Low RoB | Low RoB | Low RoB | Unclear | Low RoB | Low RoB | Low RoB | Low RoB | Low RoB | Low RoB | Low RoB | High RoB |
| Adela Arpitha et al, 2020 | Low RoB | Low RoB | Low RoB | Unclear | Unclear | Unclear | Low RoB | Unclear | Low RoB | Unclear | Unclear | Unclear | Unclear | Unclear | Low RoB | High RoB | Unclear | Low RoB | Low RoB | Low RoB | Unclear |
| Jin Han et al, (DCNet), 2020 | High RoB | Low RoB | Unclear | Low RoB | Low RoB | Low RoB | Unclear | High RoB | Low RoB | Unclear | Low RoB | High RoB | Low RoB | Low RoB | High RoB | Unclear | Low RoB | Unclear | Low RoB | Unclear | High RoB |
| Eren Bora Yilmaz et al, (Pre-trained U-Net Prefix), 2020 | High RoB | Low RoB | Unclear | Unclear | Low RoB | Unclear | Low RoB | High RoB | Low RoB | Unclear | High RoB | Low RoB | Unclear | High RoB | Low RoB | High RoB | High RoB | Low RoB | Unclear | Low RoB | High RoB |
| Eren Bora Yilmaz et al, (Custom CNN), 2020 | Unclear | Low RoB | Low RoB | Unclear | Unclear | Low RoB | Unclear | Unclear | Unclear | Unclear | Low RoB | Low RoB | Low RoB | Unclear | Unclear | Unclear | Low RoB | Unclear | Unclear | Unclear | Low RoB |
| H. Kim et al, (KNN = 3), 2023 | Low RoB | High RoB | High RoB | High RoB | Low RoB | High RoB | Unclear | Low RoB | High RoB | Low RoB | Low RoB | Low RoB | High RoB | Unclear | Unclear | High RoB | High RoB | High RoB | Low RoB | High RoB | High RoB |
| H. Kim et al, (KNN = 5), 2023 | High RoB | Low RoB | Unclear | Unclear | Low RoB | Low RoB | Low RoB | Unclear | Low RoB | Low RoB | High RoB | Unclear | Low RoB | Unclear | High RoB | High RoB | Unclear | Unclear | High RoB | Low RoB | High RoB |
| H. Kim et al, (KNN = 7), 2023 | Low RoB | Low RoB | Unclear | Unclear | Unclear | Low RoB | Unclear | Unclear | Low RoB | Low RoB | Unclear | Low RoB | Unclear | Low RoB | Unclear | High RoB | Low RoB | Unclear | Unclear | Low RoB | Unclear |
| H. Kim et al, (SVM), 2023 | Low RoB | Unclear | Low RoB | Unclear | Low RoB | Low RoB | Unclear | Low RoB | Low RoB | Unclear | Unclear | Unclear | Low RoB | Unclear | Unclear | Unclear | Unclear | Unclear | Unclear | Unclear | Low RoB |
| Joeri Nicolaes et al (3D CNN), 2019 | Low RoB | Unclear | High RoB | Low RoB | Unclear | High RoB | Unclear | Low RoB | Low RoB | Low RoB | Low RoB | Low RoB | High RoB | Unclear | Unclear | Low RoB | High RoB | Low RoB | High RoB | Unclear | High RoB |
| Sankaran Iyer et al, (DRL, IL), 2020 | Low RoB | Low RoB | Low RoB | Unclear | Unclear | Unclear | Low RoB | Unclear | Low RoB | Unclear | Low RoB | Unclear | Unclear | Unclear | Low RoB | Low RoB | Unclear | Low RoB | Unclear | High RoB | Unclear |
| David Chettrit et al, (3D CNN with sequence to sequence architecture), 2020 | Low RoB | Unclear | Low RoB | Unclear | Low RoB | Low RoB | Low RoB | Unclear | Unclear | Low RoB | Unclear | Unclear | Unclear | Low RoB | Unclear | Low RoB | Unclear | Low RoB | Unclear | High RoB | Unclear |
| Amir Bar et al, (CNN), 2017 | Low RoB | Unclear | Unclear | Low RoB | Unclear | Unclear | Low RoB | Unclear | Unclear | Low RoB | Low RoB | Low RoB | Unclear | Unclear | Unclear | Unclear | Low RoB | Unclear | High RoB | Unclear | Unclear |
| Amir Bar et al, (RNN), 2017 | Unclear | Unclear | Unclear | Low RoB | Low RoB | Unclear | Low RoB | Low RoB | Low RoB | Unclear | Unclear | Unclear | Low RoB | Unclear | Unclear | Unclear | Low RoB | Low RoB | High RoB | Low RoB | Unclear |
| Charmae B. Antonio et al, (ResNet-152), 2018 | Unclear | Low RoB | High RoB | Low RoB | Unclear | High RoB | Unclear | Low RoB | High RoB | Unclear | Low RoB | Unclear | Unclear | High RoB | High RoB | Low RoB | Low RoB | High RoB | Unclear | Low RoB | High RoB |
| Joseph E. Burns et al, (SVM), 2017 | Low RoB | Low RoB | Low RoB | Unclear | Low RoB | Low RoB | Unclear | Low RoB | Unclear | Unclear | Low RoB | Unclear | Low RoB | Low RoB | Low RoB | Unclear | Low RoB | Low RoB | Low RoB | Unclear | Low RoB |
| Carl G. Glessgen et al, (nnU-Net, ResNet18), 2022 | Low RoB | Unclear | Low RoB | Unclear | Unclear | Unclear | Unclear | Unclear | Unclear | Low RoB | Low RoB | Unclear | Unclear | Low RoB | Unclear | Unclear | Low RoB | Low RoB | Low RoB | Unclear | Low RoB |
| John Page et al, (Zebra Medical Imaging), 2020 | Low RoB | Unclear | Low RoB | Unclear | Unclear | Unclear | Low RoB | Unclear | Low RoB | Unclear | Unclear | Unclear | Unclear | Low RoB | Low RoB | High RoB | Low RoB | Unclear | Low RoB | Low RoB | Unclear |
| Hsuan-Yu Chen et al, (ResNeXt-50), 2021 | Unclear | Unclear | Unclear | Low RoB | Low RoB | Low RoB | Unclear | Unclear | Low RoB | Unclear | Unclear | Unclear | High RoB | Low RoB | Unclear | Low RoB | Low RoB | Low RoB | Unclear | Low RoB | Unclear |
| Guillermo Sánchez Rosenberg et al, (ResNet18), 2022 | Low RoB | Low RoB | Low RoB | Low RoB | Unclear | High RoB | Unclear | Unclear | High RoB | Low RoB | Low RoB | High RoB | Low RoB | Low RoB | Unclear | High RoB | High RoB | Low RoB | Unclear | High RoB | High RoB |
| Guillermo Sánchez Rosenberg et al, (VGG16), 2022 | Unclear | Low RoB | Unclear | Unclear | Unclear | Unclear | Unclear | Unclear | Unclear | Low RoB | Unclear | Unclear | Unclear | Unclear | Low RoB | Low RoB | Low RoB | Low RoB | Unclear | High RoB | Unclear |
| Wojciech M. Glinkowski et al, 2017 | Low RoB | Unclear | Low RoB | Unclear | Low RoB | Unclear | Low RoB | Unclear | Low RoB | Unclear | Unclear | Unclear | Low RoB | Low RoB | Low RoB | Unclear | Unclear | Low RoB | High RoB | Low RoB | Unclear |
| Joeri Nicolaes et al, (CNN), 2024 | Unclear | Unclear | Low RoB | Low RoB | Unclear | Unclear | Unclear | Unclear | Low RoB | Low RoB | Low RoB | Low RoB | Low RoB | High RoB | Low RoB | Unclear | Low RoB | Unclear | Low RoB | Unclear | Unclear |
| Eren Bora Yilmaz et al, (fNet), 2021 | Low RoB | Unclear | Unclear | Low RoB | Unclear | Unclear | Low RoB | Low RoB | Unclear | Low RoB | Low RoB | Unclear | Low RoB | Low RoB | Unclear | Low RoB | Low RoB | Low RoB | Unclear | Low RoB | Low RoB |
| Paul A. Bromiley et al, (k-nearest neighbours), 2018 | Unclear | Unclear | Low RoB | Low RoB | Unclear | Unclear | Unclear | Low RoB | Unclear | Low RoB | Unclear | Unclear | Low RoB | Unclear | Low RoB | Unclear | Unclear | Unclear | Unclear | Unclear | Low RoB |
| Nithin Kolanu et al, (Zebra Medical Vision), 2020 | Unclear | Low RoB | Low RoB | Unclear | Unclear | Low RoB | Low RoB | Low RoB | Low RoB | Low RoB | Unclear | Unclear | High RoB | Low RoB | Low RoB | Unclear | Low RoB | Low RoB | Low RoB | Unclear | Unclear |

**Supplementary Table 4:** Study characteristics of the included studies pertaining to fracture prediction in this systematic review.

| Study (year) | Clinical Utility | AI Model Architecture | Sample size | AUROC | Ground truth | Other performance measures |  |
| --- | --- | --- | --- | --- | --- | --- | --- |
| Bodden J et al. (2023)^1^ | Odds ratios for prevalent and incident VFs calculated for vBMD (volumetric bone mineral density) | Convolutional neural network framework | 420 | NS | Identified VFs by an expert in spine imaging | N/A |  |
| Cho ST et al (2023)^2^ | Prediction of progressive collapse in osteoporotic vertebral fractures | Multivariate logistic regression | 670 | 0.724 | Based on the compression rate variation >15% between initial and 1-year follow-up | Accuracy:0.731  F1/Dice:0.767  Precision:0.761 |  |
|  |  | Decision Tree |  | 0.688 |  | Accuracy:0.701  F1/Dice:0.75  Precision:0.72 |  |
|  |  | Random Forest |  | 0.727 |  | Accuracy:0.741  F1/Dice:0.785  Precision:0.748 |  |
| Chen Y et al. (2023)^3^ | Fracture recognition from CT images | Deep convolutional neural network (DCNN) | 210 | NS | Compared with manual annotation | Sensitivity:0.9235  F1/Dice:0.94  Precision:0.9683 |  |
|  |  | Artifcial detection combined with DCNN |  |  |  | Sensitivity:0.9406  F1/Dice:0.95  Precision:0.9742 |  |
| Park T et al. (2022)^4^ | Segmentation of fractured vertebral bodies on CT | Convolutional Neural Network (CNN) | 59 | 0.83 | Manually performed by expert image analysts and approved by a board-certified musculoskeletal radiologist. | Sensitivity:0.8  Specificity:0.72  Accuracy:0.76  PPV:0.75  NPV:0.78 |  |
| Ma Y et al. (2023)^5^ | Probability of new fractures occurring after surgery. | Logistic regression | 529 | 0.898 | Radiologists’ judgement | Sensitivity:0.831  Specificity:0.965 |  |
|  |  | Random Forest |  | 0.94 |  | Sensitivity:0.966  Specificity:0.913 |  |
|  |  | Gradient boosting machine |  | 0.91 |  | Sensitivity:0.898  Specificity:0.922 |  |
|  |  | Decision tree |  | 0.842 |  | Sensitivity:0.779  Specificity:0.904 |  |
|  |  | Support vector machine |  | 0.902 |  | Sensitivity:0.856  Specificity:0.948 |  |
|  |  | Neural network |  | 0.923 |  | Sensitivity:0.907  Specificity:0.939 |  |
|  |  | Regularized discriminant analysis |  | 0.915 |  | Sensitivity:0.881  Specificity:0.947 |  |
| Gui C. et al. (2022)^6^ | Prediction of VCF within 1 year after SBRT (Stereotactic Body Radiation Therapy) | Random forest classification models - SINS components | 85 | 0.579 | Verified VCF within 1 year post-SBRT | Sensitivity:0.956  Specificity:0.262 |  |
|  |  | Random forest classification models - Selected clinical features alone |  | 0.795 |  | Sensitivity:0.844  Specificity:0.747 |  |
|  |  | Random forest classification models - Selected CT & T1W MR features |  | 0.872 |  | Sensitivity:0.956  Specificity:0.719 |  |
|  |  | Random forest classification models - Selected CT, T1W MR, & clinical features |  | 0.878 |  | Sensitivity:0.844  Specificity:0.8 |  |
| Yunji Seol et al. (2023)^7^ | Prediction of vertebral compression fracture (VCF) occurrence. | Gradient boosting was chosen as the optimal machine learning model. Used Clinical features only | 114 | 0.746 | Based on follow-up examinations for vertebral compression fractures (VCFs) after SBRT. | Sensitivity:0.629  Accuracy:0.788  F1/Dice:0.618  Precision:0.611 |  |
|  |  | Gradient boosting was chosen as the optimal machine learning model. Used Clinical and radiomics features |  | 0.764 |  | Sensitivity:0.564  Accuracy:0.818  F1/Dice:0.573  Precision:0.6 |  |
|  |  | Gradient boosting was chosen as the optimal machine learning model. Used Radiomics and dosimetric features |  | 0.854 |  | Sensitivity:0.755  Accuracy:0.829  F1/Dice:0.65  Precision:0.625 |  |
|  |  | Gradient boosting was chosen as the optimal machine learning model. Used Radiomics and dosimetric and clinical features |  | 0.871 |  | Sensitivity:0.564  Accuracy:0.818  F1/Dice:0.573  Precision:0.6 |  |
| Hu X et al. (2022)^8^ | Predicting subsequent OVCF. | Convolutional Neural Network (CNN) using the Xception module in first OVCF | 103 | 0.883 | Confirmed by MRI or bone emission computed tomography scan. | Accuracy:0.839  AUPRC:0.71 |  |
|  |  |  |  |  |  |  |  |
| Kong HS. et al. (2020)^9^ | Osteoporotic fracture prediction | Convolutional Neural Network (CNN) with key-point detection | 1595 | 0.72 | NS | Sensitivity:0.61  Specificity:0.77 |  |
|  |  | CNN with Segmented Images |  | 0.8 |  | Sensitivity:0.74  Specificity:0.77 |  |
|  |  | Naïve-Bayes Model |  | 0.72 |  | Sensitivity:0.65  Specificity:0.77 |  |

**Supplementary Table 5:** Study characteristics of the included studies pertaining to fracture detection in this systematic review.

| Study (year) | Clinical Utility | AI Model Architecture (Internal/external validation) | Sample size | AUROC | Ground truth | Other performance measures |
| --- | --- | --- | --- | --- | --- | --- |
| Shen L et al. (2023)^10^ | Detection and grading of osteoporotic vertebral fractures | AI_OVF_SH  (Internal) | 11397 | NS | Radiologists using the Genant semiquantitative tool for fracture diagnosis and grading | Sensitivity/recall: 0.8408  Specificity:0.9725  Accuracy: 0.9741 |
|  |  | AI_OVF_SH  (External) | 1276 | NS |  | :Sensitivity/recall: 0.8335  Specificity: 0.947  Accuracy: 0.9685 |
| Hong N.  et al. (2023)^11^ | Classification of prevalent vertebral fractures | VERTE-X pVF score  (Internal) | 9276 | 0.93 | Based on algorithm-based qualitative method | Sensitivity/recall: 0.76  Specificity:0.94  Accuracy:0.91  PPV:0.74  NPV:0.95  F1/DICE:0.91  AUPRC:0.83 |
|  |  | VERTE-X pVF score  (External) | 234 | 0.92 |  | Sensitivity/recall: 0.75  Specificity:0.97  Accuracy:0.94  PPV:0.82  NPV:0.96  F1/DICE:0.78  AUPRC:0.81 |
|  |  | VERTE-X osteo score  (Internal) | 9276 | 0.85 |  | Sensitivity/recall:0.7  Specificity:0.83  Accuracy:0.77  PPV:0.73  NPV:0.8  F1/DICE:0.71  AUPRC:0.8 |
|  |  | VERTE-X osteo score  (External) | 234 | 0.83 |  | Sensitivity/recall:0.62  Specificity:0.85  Accuracy:0.72  PPV:0.85  NPV:0.63  F1/DICE:0.72  AUPRC:0.85 |
| Lama D et al. (2022)^12^ | classify vertebral bodies in MRI images into one of three classes: Normal (vertebral bodies without fracture); Benign Vertebral Compression; Malignant Vertebral Compression Fractures Fractures (VCFs) | MLP | 61 | NS | A board-certified radiologist with extensive experience in musculoskeletal radiology. | Sensitivity/recall:0.586  Specificity:0.803  F1/Dice:0.57  AUPRC:0.95 |
|  |  | CNN manually optimised |  | NS |  | Sensitivity/recall:0.766  Specificity:0.893  F1/Dice:0.76  AUPRC:0.94 |
|  |  | CNN manually optimised with data augmentation |  | NS |  | Sensitivity/recall:0.753  Specificity:0.893  F1/Dice:0.766  AUPRC:0.9 |
|  |  | Pre-trained CNN |  | NS |  | Sensitivity/recall:0.826  Specificity:0.926  F1/Dice:0.766  AUPRC:0.97 |
|  |  | Hybrid model using pre-trained CNN |  | NS |  | Sensitivity/recall:0.826  Specificity:0.926  F1/Dice:0.876  AUPRC:0.98 |
|  |  | Hybrid model optimised by the GA |  | NS |  | Sensitivity/recall:0.7  Specificity:0.856  F1/Dice:0.7  AUPRC:0.92 |
| J.E. Small et al.  (2021)^13^ | Fracture detection on cervical spine CT | Aidoc's FDA-approved convolutional neural network (CNN) for cervical spine fracture detection on CT | 665 | NS | Established by retrospective visualisation of fractures using CT, MR imaging, and CNN output | Sensitivity:0.76  Specificity:0.97  Accuracy:0.92  TN:109  TP:505  FP:17  FN:34  PPV:87  NPV:94 |
| Yabu A. et al. (2021)^14^ | Binary classification of fresh and old fractures | Combination of VGG16, VGG19, DenseNet201, and ResNet50 | 814 | 0.949 | Mark coordinates identified by two spine surgeons | Sensitivity/recall: 0.881  Specificity:0.879  Accuracy:0.88  PPV:0.841  NPV:0.911 |
| Seo JW et al. (2021)^15^ | Measurement of vertebral compression | Multi-Scale Residual Dilated Network (MRDN) | 83 | NS | NS | MAE: 2.637 (±1.872)%  MSE: 13.985 (±24.107)%  RMSE: 3.739 (±2.187)% |
| Murata K et al. (2020)^16^ | NS | Deep Convolutional Neural Network (DCNN) | 300 | 0.91 | Diagnosis determined by spine surgeons with MRI findings | Sensitivity/recall: 0.847  Specificity:0.873  Accuracy: 0.86 |
| Chen W et al. (2021)^17^ | Identification of fresh VCFs | Deep-learning (DL) model using a convolutional neural network (CNN) architecture | 1099 | 0.8 | MRI | Sensitivity/recall: 0.8  Specificity:0.68  Accuracy: 0.74 |
| Yuan Li et al. (2021)^18^ | Binary (malignant or benign) | ResNet50 | 433 | NS | Confirmed by either biopsy-proven cancer or known history of primary tumor with progressive disease for malignant cases, and no known cancer history with stable disease follow-up for benign cases. | Sensitivity/recall: 0.95 (per-patient), 0.90 (per-slice)  Specificity:0.80 (per-patient and per-slice)  Accuracy: 0.88 (per-patient), 0.85 (per-slice) |
| Kim DH et al. (2021)^19^ | Segmentation of lateral vertebral images for VCR measurement. | Multi Dilated Recurrent Residual U-Net (MDR2U-net). | 339 | 0.987 | Manually segmented vertebral area data by spine specialists. | Sensitivity/recall:0.937  Specificity:0.995  Accuracy:0.992  F1/Dice:0.929  AUPRC:0.916 |
| Golla A et al. (2023)^20^ | Identification of cervical spinal fractures | U-Net | 195 | NS | Identified and annotated by radiologists at a tertiary trauma center | Sensitivity/recall:0.872 |
| Chen H et al. (2021)^21^ | Probability of vertebral fracture | Deep Convolutional Neural Network (DCNN) | 1306 | 0.72 | Diagnosis from registry and supporting images reviewed by a radiologist and a spine surgeon. | Sensitivity/recall: 0.7381  Specificity:0.7302  Accuracy: 0.7359 |
| Biamonte E. et al. (2022)^22^ | Association with fragility vertebral fractures | Linear Support Vector Machine (LSVM) | 240 | 0.789 | Diagnosed vertebral fractures | Sensitivity/recall: 0.78  Specificity:0.696  Accuracy: 0.717 |
| Tomita N. (2018)^23^ | Probability value for the diagnosis of osteoporotic vertebral fracture on a CT scan | Combination of CNN and LSTM | 1432 | 0.9 | Established through semiquantitative and quantitative re-evaluation by domain-expert radiologists | Sensitivity/recall: 0.852  Specificity:0.958  Accuracy: 0.892  F1/Dice:0.908  Precision:0.972 |
| Xu F. et al. (2023)^24^ | Classification of VCFs into acute, chronic, and pathological types. | Deep learning model based on the ResNet-18 architecture.  (External) | 97 | NS | Established through MRI diagnosis and positron emission tomography (PET) or histopathological results. | Sensitivity:0.748  Specificity:0.874  Accuracy:0.832  PPV:0.748  NPV:0.874  F1/Dice:0.752  Precision:0.757 |
|  |  |  | 147 | NS |  | Sensitivity:0.636  Specificity:0.818  Accuracy:0.757  PPV:0.636  NPV:0.818  F1/Dice:0.652  Precision:0.668 |
|  |  |  | 143 | NS |  | Sensitivity:0.688  Specificity:0.844  Accuracy:0.792  PPV:0.687  NPV:0.844  F1/Dice:0.687  Precision:0.686 |
| Lee Y. et al.  (2023)^25^ | Location and prediction of likelihood of Vertebral Compression Fractures (VCFs) | ALiGN, an improved Faster R-CNN model  (Internal) | 5333 | NS | Diagnosis based annotations by neurosurgeons | Sensitivity:0.9867  Specificity:0.9957 |
| Voter AF et al. (2021)^26^ | Positive or negative for CSFx | Aidoc  (Internal) | 1904 | NS | Final neuroradiologist interpretation | Sensitivity:0.549  Specificity:0.941  TP:67  FP:106  FN:55  PPV:0.387  NPV:0.968 |
| Cai J et al.  (2023)^27^ | Probability of NVF | Combined-LR  (Internal) | 42 | NS | Determined by post-operative spinal MRI to confirm NVF occurrence. | Sensitivity:0.57  Specificity:0.76  Accuracy:0.67  PPV:0.76  NPV:0.64 |
|  | Probability of NVF | Combined-RF  (Internal) |  | NS | Determined by post-operative spinal MRI to confirm NVF occurrence. | Sensitivity:0.67  Specificity:0.81  Accuracy:0.74  PPV:0.81  NPV:0.71 |
|  | Probability of NVF | Combined-SVM 0  (Internal) |  | NS | Determined by post-operative spinal MRI to confirm NVF occurrence. | Sensitivity:0.67  Specificity:0.9  Accuracy:0.79  PPV:0.9  NPV:0.73 |
|  | Probability of NVF | Combined-XGBoost  (Internal) |  | NS | Determined by post-operative spinal MRI to confirm NVF occurrence. | Sensitivity:0.89  Specificity:0.87  Accuracy:0.88  PPV:0.87  NPV:0.91 |
| Monchka BA  et al. (2021)^28^ | Classification as fracture or non-fracture | Single-energy  (Internal) | 12742 | 0.92 | Classification by imaging specialists using the modified algorithm-based qualitative (mABQ) method | Sensitivity:0.768  Specificity:0.906  Accuracy:0.886  PPV:0.629  NPV:0.954 |
|  |  | Composite  (Internal) |  | 0.94 |  | Sensitivity:0.882  Specificity:0.923  Accuracy:0.906  PPV:0.686  NPV:0.962 |
|  |  | Dual-energy  (Internal) |  | 0.58 |  | Sensitivity:0.474  Specificity:0.631  Accuracy:0.604  PPV:0.207  NPV:0.855 |
|  |  | Single-energy  (Internal) |  | 0.58 |  | Sensitivity:0.303  Specificity:0.786  Accuracy:0.704  PPV:0.223  NPV:0.847 |
|  |  | Composite  (Internal) |  | 0.95 |  | Sensitivity:0.824  Specificity:0.943  Accuracy:0.923  PPV:0.745  NPV:0.963 |
|  |  | Dual-energy  (Internal) |  | 0.94 |  | Sensitivity:0.879  Specificity:0.874  Accuracy:0.875  PPV:0.587  NPV:0.973 |
| Perez‑Diaz M. (2023)^29^ | Differentiation between benign and malignant vertebral compression fractures | Machine Learning (Gaussian Naïve Bayes algorithm) | 479 | 0.86 | Based on biopsy and pathological results | Accuracy: 0.8761 |
| Li YC et al. (2021)^30^ | Detection of vertebral fractures | Deep-learning ensemble model | 941 | 0.919 for Grade 1, 0.989 for Grade 2, and 0.990 for Grade 3 fractures | Interpretation of the CT or MRI reports by a spine surgeon and a radiologist independently | Sensitivity:0.91  Specificity:0.93  Accuracy:0.92 |
| Yoda T et al. (2022)^31^ | Classification of osteoporotic vertebral fractures and malignant vertebral compression fractures | STIR CNN | 97 | 0.967 | accuracy, sensitivity, and specificity of the diagnosis made by the CNN and three spine surgeons | Sensitivity:0.925  Specificity:0.949  Accuracy:0.938 |
|  |  | T1WI CNN |  | 0.984 |  | Sensitivity:0.981  Specificity:0.949  Accuracy:0.964 |
| Monchka BA, et al. (2022)^32^ | Binary classification (fracture or non-fracture) | Maximum ensemble CNN OsteoLaus Study data set Hologic VFA type | 22281 | 0.98 | Expert review using modified algorithm-based qualitative (mABQ) method | Sensitivity:0.919  Specificity:0.99  Accuracy:0.954  PPV:0.81  NPV:0.996  F1/Dice:0.861 |
|  |  | Maximum ensemble CNN Manitoba data set GE dual-energy VFA type |  | 0.95 |  | Sensitivity:0893.  Specificity:0.881  Accuracy:0.887  PPV:0.604  NPV:0.976  F1/Dice:0.72 |
|  |  | Maximum ensemble CNN Manitoba data set GE single-energy VFA type |  | 0.95 |  | Sensitivity:0.898  Specificity:0.857  Accuracy:0.877  PPV:0.561  NPV:0.976  F1/Dice:0.69 |
|  |  | Mean ensemble CNN OsteoLaus Study data set Hologic VFA type |  | 0.98 |  | Sensitivity:0.73  Specificity:1  Accuracy:0.865  PPV:1  NPV:0.987  F1/Dice:0.844 |
|  |  | Mean ensemble CNN Manitoba data set GE dual-energy VFA type |  | 0.95 |  | Sensitivity:0.819  Specificity:0.966  Accuracy:0.893  PPV:0.832  NPV:0.963  F1/Dice:0.825 |
|  |  | Mean ensemble CNN Manitoba data set GE single-energy VFA type |  | 0.95 |  | Sensitivity:0.805  Specificity:0.964  Accuracy:0.885  PPV:0.82  NPV:0.96  F1/Dice:0.812 |
| Germann C et al. (2023)^33^ | Insufficiency fracture detection and vertebral body measurements | Deep Convolutional Neural Network (DCNN)  (Internal) | 200 | NS | Determined by medical annotators and radiologists using a medical annotation platform. | Sensitivity:0.941  Specificity:0.969  Accuracy:0.968  TP:224  TN:738  FP:24  FN:14 |
| Zhang J et al. (2023)^34^ | Classification of vertebral body fractures according to AO classification | Multistage deep learning system (multistage AO system)  (Internal) | 1217 | 0.993 | Marked and classified by 2 junior radiology residents and a director radiologist according to the type A standard in the AO classification. | Sensitivity:0.9523  Specificity:0.9835  Accuracy:0.9793 |
| Wu-Gen Li et al. (2023)^35^ | Predictive model for detecting OVFs | Support Vector Machine (SVM) - Sagittal Imaging | 128 | 0.805 | MRI confirmation of bone marrow edema and classification based on the Genant semi-quantitative system | Sensitivity:0.538  Specificity:1  Accuracy:0.769 |
|  |  | Logistic Regression (LR) - Sagittal Imaging |  | 0.882 |  | Sensitivity:0.846  Specificity:0.846  Accuracy:0.846 |
|  |  | Bayes - Sagittal Imaging |  | 0.834 |  | Sensitivity:0.923  Specificity:0.692  Accuracy:0.808 |
|  |  | Support Vector Machine (SVM) - Coronal Imaging |  | 0.692 |  | Sensitivity:0.615  Specificity:0.917  Accuracy:0.731 |
|  |  | Logistic Regression (LR) - Coronal Imaging |  | 0.775 |  | Sensitivity:0.462  Specificity:1  Accuracy:0.731 |
|  |  | Bayes - Coronal Imaging |  | 0.68 |  | Sensitivity:0.923  Specificity:0.462  Accuracy:0.692 |
| Chiari-Correia NS et al. (2023)^36^ | Classification of vertebral compression fractures as benign or malignant. | Multilayer perceptron neural network with a back-propagation algorithm. | 100 | 0.97 | Established through a combination of medical records, biopsies for malignant fractures, DEXA scans, and imaging for benign fractures. | Sensitivity:0.993  Specificity:0.993  Accuracy:0.993  TP:14  TN:14  FP:1  FN:1 |
| Eller-Vainicher C et al. (2011)^37^ | Prediction of the presence of morphometric vertebral fractures (SDI≥1 or SDI≥5 vs. SDI = 0). | CNN identifying patients with SDI≥1 | 372 | 0.714 | Determined based on clinical and morphometric criteria. | Sensitivity:0.725  Specificity:0.785  Accuracy:0.755 |
|  |  | CNN identifying patients with SDI≤1 |  | 0.823 |  | Sensitivity:0.748  Specificity:0.878  Accuracy:0.813 |
| Liu B et al. (2023)^38^ | Classification of VCFs as benign or malignant | TSCCN - T1WIT1-weighted images | 209 | 0.992 | Comparison with radiologists’ assessments and histological confirmation for malignant cases | Sensitivity:0.907  Specificity:0.984  Accuracy:0.952 |
|  |  | TSCCN -T2WI-FS T2-weighted fat suppression |  | 0.917 |  | Sensitivity:0.849  Specificity:0.943  Accuracy:0.904 |
|  |  | TSCCN - T1WI/T2WI-FS |  | 0.982 |  | Sensitivity:0.919  Specificity:0.992  Accuracy:0.962 |
| Muehlematter UJ et al. (2019)^39^ | Classification of vertebral bones as at risk or not for insufficiency fractures. | SVM | 58 | 0.97 for SVM in differentiating patients at risk for insufficiency fractures; 0.64 for distinguishing unstable/stable vertebrae within subjects. | Based on the presence of newly occurred osteoporotic vertebral compression fractures assessed by CT scans. | Accuracy:0.97 |
| Nicolaes J et al. (2023)^40^ | Estimated semiquantitative (SQ) grade for each identified vertebra in a scan | SQ23 (normal/mild versus moderate/severe) - Subject level | 1943 | 0.876 | Reference standard readings using the Genant semiquantitative (SQ) grading | Sensitivity:0.808  Specificity:0.945  Accuracy:0.92  PPV:0.725  NPV:0.965 |
|  |  | SQ23 (normal/mild versus moderate/severe) - Vertebral level |  | 0.763 |  | Sensitivity:0.532  Specificity:0.993  Accuracy:0.98  PPV:0.667  NPV:0.987 |
|  |  | SQ123 (normal versus mild/moderate/severe) outcome - Subject level |  | 0.815 |  | Sensitivity:0.757  Specificity:0.873  Accuracy:0.85  PPV:0.612  NPV:0.931 |
|  |  | SQ123 (normal versus mild/moderate/severe) outcome - Vertebral level |  | 0.728 |  | Sensitivity:0.47  Specificity:0.987  Accuracy:0.96  PPV:0.616  NPV:0.977 |
| Nicolaes J et al. (2024)^41^ | List of vertebrae with a VF grade associated with every level identified in the scan | SQ23 (normal/mild versus moderate/severe) - Subject level | 4810 | 0.938 | Reference standard readings | Sensitivity:0.944  Specificity:0.932  Accuracy:0.933  PPV:0.675  NPV:0.991 |
|  |  | SQ23 (normal/mild versus moderate/severe) - Vertebral level |  | 0.932 |  | Sensitivity:0.874  Specificity:0.99  Accuracy:0.988  PPV:0.632  NPV:0.998 |
|  |  | SQ123 (normal versus mild/moderate/severe) outcome - Subject level |  | 0.781 |  | Sensitivity:0.626  Specificity:0.935  Accuracy:0.831  PPV:0.831  NPV:0.831 |
|  |  | SQ123 (normal versus mild/moderate/severe) outcome - Vertebral level |  | 0.783 |  | Sensitivity:0.576  Specificity:0.99  Accuracy:0.967  PPV:0.762  NPV:0.976 |
| Duan S et al. (2023)^42^ | Classification of VCFs into OVCFs and MVCFs | Deep Learning (DL) Model | 280 | 0.89 | Clinical diagnosis confirmed by pathology | Sensitivity:0.88  Specificity:0.87  Accuracy:0.88  F1/Dice:0.86 |
|  |  | Radiomics (Rad) Model |  | 0.93 |  | Sensitivity:0.84  Specificity:0.97  Accuracy:0.91  F1/Dice:0.89 |
|  |  | Combined Deep Learning and Radiomics (DL_Rad) Model |  | 0.97 |  | Sensitivity:0.92  Specificity:0.94  Accuracy:0.93  F1/Dice:0.93 |
| Dong Q et al. (2023)^43^ | Probability of OCF presence. | Ensemble Averaging (Task 2, Local-m2ABQ test) | 1790 | 0.948 | Annotated using the modified-2 algorithm-based qualitative (m2ABQ) criteria for the local dataset and a modification of the Genant semi-quantitative (mSQ) criteria for the MrOS dataset. | Sensitivity:0.545  Specificity:0.997  Accuracy:0.977  PPV:0.898  NPV:0.979  F1/Dice:0.671 |
|  |  | Ensemble Averaging (Task 2, MrOS-m2ABQ test) |  | 0.936 |  | Sensitivity:0.478  Specificity:0.996  Accuracy:0.925  PPV:0.948  NPV:0.924 |
|  |  | Ensemble Averaging (Task 3, Local-m2ABQ test) |  | 0.955 |  | Sensitivity:0.539  Specificity:0.997  Accuracy:0.977  PPV:0.894  NPV:0.979  F1/Dice:0.671 |
| Al-Helo S. et al. (2012)^44^ | Diagnosis of vertebra wedge compression fracture | Neural Network | 50 | NS | Diagnosis from clinical reports | Accuracy:0.932 |
|  | Diagnosis of vertebra wedge compression fracture | Neural Network | 50 | NS | Diagnosis from clinical reports | Sensitivity:0.875  Specificity:0.991  Accuracy:0.98 |
| Thawait SK et al. (2013)^45^ | Discrimination of benign from malignant VCFs | Logistic Regression | 128 | 0.762 | Biopsy or follow-up imaging | N/A |
|  |  | Classification Tree |  | 0.76 |  |  |
|  |  | Support Vector Machine |  | 0.68 |  |  |
|  |  | Neural Network |  | 0.74 |  |  |
| Choi E et al. (2023)^46^ | Vertebral-level VCF classification probabilities | MULAN (MUlti-LAbel classification Network with the image-level labelled data) | 1002 | NS | Genant’s semi-quantitative classification method by radiologists | Sensitivity:0.77  Specificity:0.981  Accuracy:0.956 |
|  |  | Vertebral-level supervised |  |  |  | Sensitivity:0.705  Accuracy:0.952 |
| Wang X et al. (2023)^47^ | Probability of VCFs being malignant or benign. | Radiomics Model | 144 | 0.962 | Confirmed by histopathology or clinical follow-up examination. | Sensitivity:0.917  Specificity:0.95  Accuracy:0.932  PPV:0.957  NPV:0.905 |
|  |  | Clinical-Radiomics Model |  | 0.948 |  | Sensitivity:0.958  Specificity:0.9  Accuracy:0.932  PPV:0.92  NPV:0.947 |
| Page JH et al. (2023)^48^ | Positive/negative finding for VCF on CT scans. | VCF detection algorithm by Zebra Medical Imaging for diagnosing any VCF | 1087 | NS | Diagnosis of board-certified neuroradiologists used as the reference standard. | Sensitivity:0.66  Specificity:0.9 |
|  |  | VCF detection algorithm by Zebra Medical Imaging for diagnosing moderate/severe VCF |  |  |  | Sensitivity:0.78  Specificity:0.87 |
| Silberstein J et al. (2023)^49^ | Identification of OVFs with a percentage indicating the likelihood of a true fracture. | Ofeye 1.0 | 510 | NS | Consultant radiologist's findings served as the reference standard. | Sensitivity:0.49  Specificity:0.928  Accuracy:0.803  TP:73  TN:335  FP:26  FN:76  PPV:0.737  NPV:0.815 |
| Wang YN et al. (2024)^50^ | Classification of vertebrae as fractured or normal | yolov7 | 716 | 0.92 | Based on radiologist diagnoses and labelled as either fresh fractures or normal vertebrae | Sensitivity:0.93  Specificity:0.92  Accuracy:0.924 |
|  |  | ResNet50 |  | 0.98 |  | Sensitivity:0.97  Specificity:0.98  Accuracy:0.976 |
| Xiao, B. et al. (2022)^51^ | Probability of CVF detection | Ofeye 1.0. | 6674 | NS | Established by experienced radiologist readers and previous osteoporotic vertebral fracture epidemiological studies. | Sensitivity:0.86  Specificity:0.971  Accuracy:0.939  TP:185  TN:476  FP:13  FN:30 |
| Cheng LW et al. (2024)^52^ | Classification of vertebral bodies as normal, compression fracture, burst fracture, or others. | RF | 390 | NS | The labeled images were verified against CT or MRI images by an experienced orthopedic doctor. | Accuracy:0.98 |
|  |  | KNN |  |  |  | Accuracy:0.89 |
|  |  | SVM |  |  |  | Accuracy:0.89 |
|  |  | MLP |  |  |  | Accuracy:0.89 |
|  |  | XGBoost |  |  |  | Accuracy:0.96 |
| Ono Y et al. (2023)^53^ | The probability that the input image is normal, old, or fresh OLVF. | Resnet-50, DenseNet-161, and ResNeXt-50 | 4143 | 0.768 | Visual evaluation by two radiologists. | Sensitivity:0.674  Specificity:0.866  Accuracy:0.867 |
| Gadu SR et al. (2023)^54^ | Segmented spine X-ray images with effective results. | Hybrid UDA Net architecture | NS | NS | NS | Specificity:0.99  Accuracy:0.9841  F1/Dice:0.9675 |
| Zhang H et al. (2023)^55^ | Differentiation of benign vs. malignant indistinguishable VCFs | GNB  (External) | 103 | 0.84 | Based on biopsy and pathological results | Sensitivity:0.9138  Specificity:0.7333  Accuracy:0.835  F1/Dice:0.83 |
|  |  | KNN  (External) |  | 0.6 |  | Sensitivity:0.931  Specificity:0.1778  Accuracy:0.6019  F1/Dice:0.53 |
|  |  | LR  (External) |  | 0.72 |  | Sensitivity:0.9483  Specificity:0.6  Accuracy:0.7961  F1/Dice:0.78 |
|  |  | MLP  (External) |  | 0.71 |  | Sensitivity:0.9138  Specificity:0.6889  Accuracy:0.8155  F1/Dice:0.81 |
|  |  | DT  (External) |  | 0.73 |  | Sensitivity:0.7931  Specificity:0.7111  Accuracy:0.7573  F1/Dice:0.75 |
|  |  | LD  (External) |  | 0.72 |  | Sensitivity:0.7414  Specificity:0.6  Accuracy:0.6796  F1/Dice:0.68 |
|  |  | GB  (External) |  | 0.79 |  | Sensitivity:0.9643  Specificity:0.5714  Accuracy:0.781  F1/Dice:0.74 |
| Ryu SM et al. (2023)^56^ | Classifies pixels into four classes (background, VCF, other normal vertebral bodies, and sacrum) for VCF detection and four classes for vertebral level detection. | ResNet101 - multi-task (ours) | 2273. | 0.953 | Based on radiological reports indicating normal findings or fractures at some vertebral level, using a 4-point trapezoidal shape. | Sensitivity:0.944  Specificity:0.917  Accuracy:0.929  F1/Dice:0.923 |
| Yilmaz EB et al. (2023)^57^ | The presence of at least 2 mild or 1 moderate fracture. | A multi-head feed-forward convolutional neural network. | 159 | NS | Annotations including the fracture grade and vertebra centroids were provided by an expert radiologist using SpineAnalyzer. | Sensitivity:0.9  Specificity:0.87 |
| Iyer S. et al. (2023)^58^ | Vertebral compression fracture detection | 3-layered CNN  (External) | 308 | NS | Radiology reports and Genant's criteria | Sensitivity:0.8216  Specificity:0.5995  Accuracy:0.7172  F1/Dice:0.7403 |
|  |  | 6-layered CNN  (External) |  |  |  | Sensitivity:0.8485  Specificity:0.7898  Accuracy:0.8222  F1/Dice:0.8267 |
|  |  | VGG16  (External) |  |  |  | Sensitivity:0.7669  Specificity:0.6495  Accuracy:0.7162  F1/Dice:0.7209 |
|  |  | ResNet50  (External) |  |  |  | Sensitivity:0.6938  Specificity:0.7911  Accuracy:0.7455  F1/Dice:0.7292 |
| Bhavya MBS et al. (2022)^59^ | Probability of fracture for each of the seven cervical vertebrae (C1 to C7) and overall fracture probability. | Deep Convolution Neural Network (CNN). | 2019 | NS | Based on radiologist observations and performance on the test set. | Accuracy:0.93 |
| Feng S et al. (2021)^60^ | Classifies vertebrae into normal, benign VCFs, and malignant VCFs. | Two-Stream Compare and Contrast Network (TSCCN) | 239 | 0.9835 | NS | Sensitivity:0.9256  Specificity:0.9629 |
| Arpitha A et al (2020)^61^ | Classification of vertebral bodies into normal or fractured, benign or malignant. | Computer aided design that incorporates various Machine Learning techniques at different stages | 850 | NS | Diagnosed results of consensus between two in-house observers and one external observer. | Accuracy: (96.07% for Case 1, 93.23% for Case 2, and 92.3% for Case 3). |
| Han J et al (2020)^62^ | Classification of spinal fracture severity. | Deformable Cascade Network (DCNet) | 362 | NS | Based on the hollow area of the spinal canal in the CT image and the corresponding fracture grade. | Precision: 0.917 |
| Yilmaz EB et al. (2020)^63^ | Fracture status score between 0 and 1, Genant score, and deformity percentages. | Pre-trained U-Net Prefix | 159 | 0.939 | Annotations provided by radiologists using SpineAnalyzerTM. | Sensitivity:0.824  Specficity0.912  Precision: 0.703 |
|  |  | Custom CNN |  | 0.989 |  | Sensitivity:0.906  Specficity0.958  Precision: 0.907 |
| Kim H. et al. (2023)^64^ | Probability of vertebral compression fracture presence. | KNN = 3 | NS | NS | Based on radiologist assessment. | Sensitivity:0.8  Specificity:0.9125  Accuracy:0.875 |
|  |  | KNN = 5 |  |  |  | Sensitivity:0.925  Specificity:0.833  Accuracy:0.883 |
|  |  | KNN = 7 |  |  |  | Sensitivity:0.9005  Specificity:0.8385  Accuracy:0.886 |
|  |  | SVM |  |  |  | Sensitivity:0.914  Specificity:0.85  Accuracy:0.886 |
| Nicolaes J et al. (2019)^65^ | Patient-level and vertebra-level fracture predictions | 3D Convolutional Neural Network (CNN) | 90 | 0.95 | Semi-automatically generated using radiologist readings | Sensitivity:0.905  Specificity:0.938 |
| Iyer S et al. (2020)^66^ | The presence or absence of Vertebral Compression Fractures. | A combination of Deep Reinforcement Learning, Imitation Learning, and a Convolutional Neural Network (CNN). | 127 | NS | NS | Sensitivity:0.7987  Specificity:0.8073  Accuracy:0.8 |
| Chettrit D et al. (2020)^67^ | Identification and localization of vertebral compression fractures. | Ensemble of 3D Convolutional Neural Networks (CNN) with Sequence to Sequence architecture. | 346 | NS | Determined by consensus agreement of three US Board Certified radiologists. | Sensitivity:0.822  Specificity:0.951  Accuracy:0.955 |
| Bar A et al (2017)^68^ | Binary classification of patches as normal or indicative of VCF | Convolutional Neural Network (CNN) | 1673 | NS | Determined by expert radiologists based on Genant criteria for vertebral compression | Accuracy:0.929 |
|  | Prediction of the presence of a VCF in the CT scan | Recurrent Neural Network (RNN) |  |  | Based on expert radiologist assessment | Sensitivity:0.839  Specificity:0.938  Accuracy:0.891 |
| Antonio CB et al. (2018)^69^ | Vertebra fracture classification | ResNet-152 | 15 | NS | Semi-quantitative vertebra fracture classification system proposed by Genant et al. | Accuracy:0.9329 |
| Burns JE et al. (2017)^70^ | Detection and classification of vertebral compression fractures, bone density measurements. | Support Vector Machine (SVM) | 150 | NS | Manual annotation by radiologists. | Sensitivity:0.957  Specificity:0.773  Accuracy:0.68  TP:201  FP:Rate of 0.29 per patient  FN:9 |
| Glessgen CG et al. (2022)^71^ | The pipeline outputs whether a vertebra is affected by a fracture. | Two-step pipeline with nnU-Net for segmentation and ResNet18 for fracture classification. | 452 | NS | Consensual ground truth obtained by two senior readers assessing fractures in the test set. | Sensitivity:0.879  Specificity:0.949  Accuracy:0.93  F1/Dice:0.87  Precision:0.869  TP:80  TN:227  FP:12  FN:11  PPV:0.869  NPV:0.953 |
| Page J et al. (2020)^72^ | Diagnosis of vertebral compression fractures (VCFs) | Zebra Medical Imaging's VCF detection algorithm | 1087 | NS | Determination of presence and severity of VCF by neuroradiologists used as the reference standard. | Sensitivity:0.66  Specificity:0.9 |
| Chen HY et al. (2021)^73^ | Detection and localization of vertebral fractures | ResNeXt-50 (Deep Convolutional Neural Network) | 1306 | 0.72 | The diagnosis was finalized when agreement was reached between a radiologist and a spine surgeon based on the semi-quantitative method of Genant for classifying vertebral fractures. | Sensitivity:0.7381  Specificity:0.7302  Accuracy:0.7359 |
| Rosenberg GS et al. (2022)^74^ | Classification of vertebrae as “fracture” or “no fracture” | ResNet18 | 630 | NS | Established by a panel of expert spinal surgeons | Sensitivity:0.91  Specificity:0.89  Accuracy:0.88  NPV:0.89 |
|  |  | VGG16 |  |  |  | Sensitivity:0.9  Specificity:0.83  Accuracy:0.86  NPV:0.89 |
| Wojciech M. et al. (2017)^75^ | Identification of vertebral fractures using automated and enhanced imaging techniques. | Software-assisted semi-automated quantitative vertebral morphometry using a model-based shape recognition algorithm (SpineAnalyzer, Optasia Medical, Cheadle, UK). | 250 | NS | Manually adjusted morphometry was considered the standard for evaluating software-assisted vertebral fracture detection. | Sensitivity:<.17  Accuracy: Approx. 86% |
| Nicolaes J et al. (2024)^76^ | List of vertebrae with VF grade for each identified level in the scan. | Convolutional Neural Network (CNN) | 4810 | 0.94 | Reference standard readings were determined by radiologists using the semiquantitative (SQ) Genant classification. | Sensitivity:0.94  Specificity:0.93  Accuracy:0.93 |
| Yilmaz EB et al.  (2021)^77^ | Classification of osteoporotic fracture presence | Feed-forward Convolutional Neural Network (fNet) | 145 | 0.986 | Annotated by SpineAnalyzerTM | Sensitivity:0.96  Specificity:0.905 |
| Bromiley PA et al. (2018)^78^ | 320 | k-nearest neighbours | 3220 | NS | Clinician data inputted into PACS | Sensitivity:0.8 |
| Kolanu N et al. (2020)^79^ | Presence or absence of vertebral fracture | Zebra Medical Vision | 1696 | NS | Adjudicated imaging specialist reevaluation | Sensitivity:0.54  Specificity:0.92  Accuracy:0.83  PPV:0.69  NPV:0.87 |
| Monchka BA et al. (2022)^80^ | Classification of vertebral fractures. | Convolutional Neural Network (CNN) | 22,281 | NS | OsteoLaus validation and test sets | Sensitivity:0.80  Specificity:0.997  PPV:0.941  F1/Dice:0.865 |
|  |  | Convolutional Neural Network (CNN) with active learning |  | NS |  | Sensitivity:0.919  Specificity:0.99  PPV:0.81  F1/Dice:0.861 |

# **References**

1. Ensrud, K. E. Epidemiology of fracture risk with advancing age. J. Gerontol. A Biol. Sci. Med. Sci. 68, 1236-1242 (2013).
2. Whitney, E. & Alastra, A. J. Vertebral Fracture. StatPearls [Internet]. Treasure Island (FL): StatPearls Publishing; 2024 Jan-. (2023).
3. Dong, Y. et al. Global incidence, prevalence, and disability of vertebral fractures: a systematic analysis of the global burden of disease study 2019. Spine J. **22**, 857-868 (2022).
4. Freitas, S. S. et al. Rate and circumstances of clinical vertebral fractures in older men. Osteoporos. Int. 19, 615-623 (2007).
5. Nevitt, M. C. et al. Risk factors for a first-incident radiographic vertebral fracture in women ≥65 years of age: the study of osteoporotic fractures. J. Bone Miner. Res. 20, 131-140 (2004).
6. Savage, J. W., Schroeder, G. D. & Anderson, P. A. Vertebroplasty and kyphoplasty for the treatment of osteoporotic vertebral compression fractures. J. Am. Acad. Orthop. Surg. 22, 653-664 (2014).
7. Cooper, C., Atkinson, E. J., O’Fallon, W. M. & Melton, J. L. Incidence of clinically diagnosed vertebral fractures: a population-based study in Rochester, Minnesota, 1985-1989. J. Bone Miner. Res. 7, 221-227 (2009).
8. Fink, H. A. et al. What proportion of incident radiographic vertebral deformities is clinically diagnosed and vice versa? J. Bone Miner. Res. 20, 1216-1222 (2005).
9. Ensrud, K. E. et al. Prevalent vertebral deformities predict mortality and hospitalization in older women with low bone mass. J. Am. Geriatr. Soc. 48, 241-249 (2000).
10. Ross, P. D. Clinical consequences of vertebral fractures. Am. J. Med. 103, 30S-42S; discussion 42S-43S (1997).
11. Thomas, B. Artificial intelligence: review of current and future applications in medicine. Fed. Pract. 38, (2021).
12. Davenport, T. & Kalakota, R. The potential for artificial intelligence in healthcare. Future Healthc. J. 6, 94-98 (2019).
13. Al-Antari, M. A. Artificial intelligence for medical diagnostics—existing and future AI technology! Diagnostics. 13, 688 (2023).
14. Bajwa, J., Munir, U., Nori, A. & Williams, B. Artificial intelligence in healthcare: transforming the practice of medicine. Future Healthc. J. 8, e188-e194 (2021).
15. Hardy, M. & Harvey, H. Artificial intelligence in diagnostic imaging: impact on the radiography profession. Br. J. Radiol. 93, 20190840 (2019).
16. Kurmis, A. P. & Ianunzio, J. R. Artificial intelligence in orthopedic surgery: evolution, current state and future directions. Arthroplasty. 4, (2022).
17. De, A., Sarda, A., Gupta, S. & Das, S. Use of artificial intelligence in dermatology. Indian J. Dermatol. 65, 352 (2020).
18. Tama, B. A., Kim, D. H., Kim, G., Kim, S. W. & Lee, S. Recent advances in the application of artificial intelligence in otorhinolaryngology-head and neck surgery. Clin. Exp. Otorhinolaryngol. 13, 326-339 (2020).
19. Shen, L. et al. Using artificial intelligence to diagnose osteoporotic vertebral fractures on plain radiographs. J. Bone Miner. Res. (2023).
20. Hong, N. et al. Deep-learning-based detection of vertebral fracture and osteoporosis using lateral spine X-ray radiography. J. Bone Miner. Res. 38, 887-895 (2023).
21. Wolff, R. F. et al. PROBAST: a tool to assess the risk of bias and applicability of prediction model studies. Ann. Intern. Med. 170, 51-58 (2019).
22. Hosmer, D. W. & Lemeshow, S. Applied Logistic Regression. 2nd edn, 162-164 (2000)
23. Collins, G. S., Reitsma, J. B., Altman, D. G. & Moons, K. G. Transparent reporting of a multivariable prediction model for individual prognosis or diagnosis (TRIPOD): the TRIPOD statement. BMJ. 350, g7594 (2015).
24. Heus, P. et al. Uniformity in measuring adherence to reporting guidelines: the example of TRIPOD for assessing completeness of reporting of prediction model studies. BMJ Open. 9, e025611 (2019).
25. Chen, Y., Sun, X., Sui, X., Li, Y. & Wang, Z. Application of bone alkaline phosphatase and 25-oxhydryl-vitamin D in diagnosis and prediction of osteoporotic vertebral compression fractures. J. Orthop. Surg. Res. 18, 739 (2023).
26. Yoon, M. A. et al. Automated segmentation of the fractured vertebrae on CT and its applicability in a radiomics model to predict fracture malignancy. Sci. Rep. 12, (2022).
27. Ma, Y., Lu, Q., Yuan, F. & Chen, H. Comparison of the effectiveness of different machine learning algorithms in predicting new fractures after PKP for osteoporotic vertebral compression fractures. J. Orthop. Surg. Res. 18, (2023).
28. Hu, X. et al. Prediction of subsequent osteoporotic vertebral compression fracture on CT radiography via deep learning. View (Beijing, China) 3, (2022).
29. Kong, S. H. et al. Development of a spine X-ray-based fracture prediction model using a deep learning algorithm. Endocrinol. Metab. 37, 674-683 (2022).
30. Yilmaz, E. B. et al. Assessing attribution maps for explaining CNN-based vertebral fracture classifiers. Lect. Notes Comput. Sci. 3, 3-12 (2020).
31. Yilmaz, E. B. et al. Automated deep learning-based detection of osteoporotic fractures in CT images. Lect. Notes Comput. Sci. 376, 376-385 (2021).
32. Monchka, B. A., Kimelman, D., Lix, L. M. & Leslie, W. D. Feasibility of a generalized convolutional neural network for automated identification of vertebral compression fractures: the Manitoba Bone Mineral Density Registry. Bone. 150, 116017 (2021).
33. Monchka, B. A. et al. Development of a manufacturer-independent convolutional neural network for the automated identification of vertebral compression fractures in vertebral fracture assessment images using active learning. Bone. 161, 116427 (2022).
34. Cho, S. T. et al. Prediction of progressive collapse in osteoporotic vertebral fractures using conventional statistics and machine learning. Spine. 48, 1535 (2023).
35. Gui, C. et al. Radiomic modeling to predict risk of vertebral compression fracture after stereotactic body radiation therapy for spinal metastases. J. Neurosurg. Spine. 36, 294-302 (2022).
36. Seol, Y. et al. Predicting vertebral compression fracture prior to spinal SBRT using radiomics from planning CT. Eur. Spine J. (2023).
37. Murata, K. et al. Artificial intelligence for the detection of vertebral fractures on plain spinal radiography. Sci. Rep. 10, (2020).
38. Chen, H. Y. et al. Application of deep learning algorithm to detect and visualize vertebral fractures on plain frontal radiographs. PLOS ONE. 16, e0245992 (2021).
39. Biamonte, E. et al. Artificial intelligence-based radiomics on computed tomography of lumbar spine in subjects with fragility vertebral fractures. J. Endocrinol. Invest. 45, 2007-2017 (2022).
40. Tomita, N., Cheung, Y. Y. & Hassanpour, S. Deep neural networks for automatic detection of osteoporotic vertebral fractures on CT scans. Comput. Biol. Med. 98, 8-15 (2018).
41. Li, Y. C. et al. Can a deep-learning model for the automated detection of vertebral fractures approach the performance level of human subspecialists? Clin. Orthop. Relat. Res. (2021).
42. Zhang, J. et al. Automated detection and classification of acute vertebral body fractures using a convolutional neural network on computed tomography. J. Bone Miner. Res. 14, (2023).
43. Li, W. G. et al. The value of radiomics-based CT combined with machine learning in the diagnosis of occult vertebral fractures. BMC Musculoskelet. Disord. 24, 819 (2023).
44. Eller-Vainicher, C. et al. Recognition of morphometric vertebral fractures by artificial neural networks: analysis from GISMO Lombardia database. PLoS ONE. 6, e27277 (2011).
45. Muehlematter, U. J. et al. Vertebral body insufficiency fractures: detection of vertebrae at risk on standard CT images using texture analysis and machine learning. Eur. Radiol. 29, 2207-2217 (2018).
46. Nicolaes, J. et al. Towards improved identification of vertebral fractures in routine computed tomography (CT) scans: development and external validation of a machine learning algorithm. J. Bone Miner. Res. 38, 1856-1866 (2023).
47. Nicolaes, J. et al. External validation of a convolutional neural network algorithm for opportunistically detecting vertebral fractures in routine CT scans. Osteoporos. Int. 35, 143-152 (2024).
48. Wang, X. et al. Value of 18F-FDG-PET/CT radiomics combined with clinical variables in the differential diagnosis of malignant and benign vertebral compression fractures. EJNMMI Res. 13, 89 (2023).
49. Nicolaes, J. et al. Detection of vertebral fractures in CT using 3D convolutional neural networks. Lect. Notes Comput. Sci. 3, 3-14 (2020).
50. Nicolaes, J. et al. External validation of a convolutional neural network algorithm for opportunistically detecting vertebral fractures in routine CT scans. Osteoporos. Int. 35, 143-152 (2024).
51. Yabu, A. et al. Using artificial intelligence to diagnose fresh osteoporotic vertebral fractures on magnetic resonance images. Spine J. (2021).
52. Yoda, T. et al. Automated differentiation between osteoporotic vertebral fracture and malignant vertebral fracture on MRI using a deep convolutional neural network. Spine. 47, E347-E352 (2022).
53. Ono, Y. et al. A deep learning-based model for classifying osteoporotic lumbar vertebral fractures on radiographs: a retrospective model development and validation study. J. Imaging. 9, 187 (2023).
54. Chen, W. et al. A deep-learning model for identifying fresh vertebral compression fractures on digital radiography. Bone. 32, 1496-1505 (2021).
55. Kim, D. H. et al. Automated vertebral segmentation and measurement of vertebral compression ratio based on deep learning in X-ray images. J. Digit. Imaging. (2021).
56. Chiari-Correia, N. S. et al. A 3D radiomics-based artificial neural network model for benign versus malignant vertebral compression fracture classification in MRI. J. Digit. Imaging. 36, 1565-1577 (2023).
57. Liu, B. et al. Benign vs malignant vertebral compression fractures with MRI: a comparison between automatic deep learning network and radiologist’s assessment. Eur. Radiol. 33, 5060-5068 (2023).
58. Duan, S. et al. Differential diagnosis of benign and malignant vertebral compression fractures: comparison and correlation of radiomics and deep learning frameworks based on spinal CT and clinical characteristics. Eur. J. Radiol. 165, 110899 (2023).
59. Dong, Q. et al. Generalizability of deep learning classification of spinal osteoporotic compression fractures on radiographs using an adaptation of the modified-2 algorithm-based qualitative criteria. Acad. Radiol. 30, 2973-2987 (2023).
60. Thawait, S. K. et al. Comparison of four prediction models to discriminate benign from malignant vertebral compression fractures according to MRI feature analysis. AJR Am. J. Roentgenol. 200, 493-502 (2013).
61. Zhang, H. et al. Differentiation of benign versus malignant indistinguishable vertebral compression fractures by different machine learning with MRI-based radiomic features. Eur. Radiol. 33, 5069-5076 (2023).
62. Ryu, S. M. et al. Diagnosis of osteoporotic vertebral compression fractures and fracture level detection using multitask learning with U-Net in lumbar spine lateral radiographs. Comput. Struct. Biotechnol. J. (2023).
63. Feng, S. et al. Two-stream compare and contrast network for vertebral compression fracture diagnosis. IEEE Trans. Med. Imaging. 40, 2496-2506 (2021).
64. Rinaldi, C. et al. The early detection of osteoporosis in a cohort of healthcare workers: is there room for a screening program? J. Clin. Endocrinol. Metab. 106, e485-e495 (2021).
65. Sözen, T., Özışık, L. & Başaran, N. Ç. An overview and management of osteoporosis. Eur. J. Rheumatol. 4, 46-56 (2017).
66. Shams, R. A., Zowghi, D. & Bano, M. AI and the quest for diversity and inclusion: a systematic literature review. AI Ethics. 4, 73-88 (2023).
67. Yin, J., Ngiam, K. Y. & Teo, H. H. Role of artificial intelligence applications in real-life clinical practice: systematic review. J. Med. Internet Res. 23, e25743 (2021).
68. Mittermaier, M., Raza, M. M. & Kvedar, J. C. Bias in AI-based models for medical applications: challenges and mitigation strategies. NPJ Digit. Med. 6, 27 (2023).
69. Marwaha, J. S. & Kvedar, J. C. Crossing the chasm from model performance to clinical impact: the need to improve implementation and evaluation of AI. NPJ Digit. Med. 5, 25 (2022).
70. Cimpeanu, T. et al. Artificial intelligence development races in heterogeneous settings. Sci. Rep. 12, 5729 (2022).
71. Meng, F. et al. AI support for accurate and fast radiological diagnosis of COVID-19: an international multicenter, multivendor CT study. Eur. Radiol. 33, 4280-4291 (2022).
72. Pauling, C. et al. Commercially available artificial intelligence tools for fracture detection: the evidence. BJR Open. 6, tzd005 (2023).
